# Investigating Pneumonia Etiology Among Refugees and the Lebanese population (PEARL): A study protocol

**DOI:** 10.12688/gatesopenres.12811.2

**Published:** 2019-06-13

**Authors:** Thomas Kesteman, Ali Ghassani, Crystel Hajjar, Valentina Picot, Marwan Osman, Zahraa Alnajjar, Florence Komurian-Pradel, Melina Messaoudi, Stéphane Pouzol, PEARL Study Group, Hicham Ghazi Soulaiman, Philippe Vanhems, Octavio Ramilo, Dolla Karam-Sarkis, Josette Najjar-Pellet, Monzer Hamze, Hubert Endtz

**Affiliations:** 1 Fondation Mérieux, Lyon, 69002, France; 2 Amel Association, Beirut, Lebanon; 3 Faculté de Pharmacie, Université Saint-Joseph, Beirut, Lebanon; 4 Laboratoire Microbiologie Santé et Environnement, Lebanese University, Tripoli, Lebanon; 5 Chtoura Hospital, Chtoura, Lebanon; 6 Al-Bashaer Medical Center, Tripoli, Lebanon; 7 Infection Control and Epidemiology Unit, Edouard Herriot Hospital, Hospices Civils de Lyon, Lyon, 69002, France; 8 Nationwide Childrens’ Hospital and the Ohio State University College of Medicine, Columbus, OH, 43205, USA; 9 Laboratoire des Agents Pathogènes, Faculté de Pharmacie, Université Saint-Joseph, Beirut, Lebanon; 10 Laboratoire Rodolphe Mérieux, Université Saint-Joseph, Beirut, Lebanon; 11 Erasmus Medical Center, Rotterdam, The Netherlands

**Keywords:** Community-Acquired Pneumonia, Lebanon, Refugees, Etiology, Case-Control Studies, Epidemiology, Prevention & Control, Risk Factors

## Abstract

**Background: **Community-acquired pneumonia (CAP), a leading cause of mortality, mainly affects children in developing countries. The harsh circumstances experienced by refugees include various factors associated with respiratory pathogen transmission, and clinical progression of CAP. Consequently, the etiology of CAP in humanitarian crisis situations may differ to that of settled populations, which would impact appropriate case management. Therefore, the Pneumonia Etiology Among Refugees and the Lebanese population (PEARL) study was initiated with the objective of identifying the causal pathogenic microorganisms in the respiratory tract of children and adults from both the refugee and host country population presenting with signs of CAP during a humanitarian crisis.

**Methods: **PEARL, a prospective, multicentric, case-control study, will be conducted at four primary healthcare facilities in Tripoli and the Bekaa valley over 15 months (including two high-transmission seasons/winters). Sociodemographic and medical data, and biological samples will be collected from at least 600 CAP cases and 600 controls. Nasopharyngeal swabs, sputum, urine and blood samples will be analyzed at five clinical pathology laboratories in Lebanon to identify the bacterial and viral etiological agents of CAP. Transcriptomic profiling of host leukocytes will be performed.

**Conclusions:** PEARL is an original observational study that will provide important new information on the etiology of pneumonia among refugees, which may improve case management, help design antimicrobial stewardship interventions, and reduce morbidity and mortality due to CAP in a humanitarian crisis.

## Introduction

Lower respiratory tract infections (LRTI) are the second leading cause of mortality worldwide, accounting for an estimated 2.8 million deaths annually, and mainly affect children in developing countries ^1^. Community-acquired pneumonia (CAP) is caused by a variety of bacteria and viruses and is mainly characterized by lobar or broncho-pneumonic changes. However, identification of the etiology of pneumonia is often difficult, and optimal prevention and treatment strategies for CAP critically depend on a full understanding of its etiology. For example, intracellular (“atypical”) bacteria ( *Chlamydophila, Mycoplasma,* etc.) require treatment with specific antibiotics, while purely viral infections do not. Furthermore, clinical exams, chest radiology and biological tests lack specificity, and blood cultures yield positive results in only 10 to 20% of cases.

Current interventions for CAP are primarily based on etiological studies conducted in the early 1980s ^2– 4^, which indicated bacteria are responsible for almost half of all cases of CAP ^5, 6^; *Streptococcus pneumoniae*, *Haemophilus influenzae* and *Staphylococcus aureus* were the most commonly identified bacteria. In children < 5-years-old, bacteria were responsible for severe forms of CAP: *S. pneumoniae* and *H. influenzae* accounted for approximately 60% of cases of severe and fatal pneumonia ^7, 8^ and *S. pneumoniae* alone accounted for 11% of mortalities overall in children < 5-years-old ^9^. However, other bacteria (e.g. *Mycoplasma pneumoniae, Chlamydophila pneumoniae, Legionella pneumophila, Bordetella pertussis,* etc. *)* may be responsible for a significant proportion of CAP ^10^. Moreover, respiratory viruses such as influenza viruses, respiratory syncytial virus (RSV) and human metapneumovirus (hMPV) are known to make significant, often seasonal, contributions to CAP. Finally, superinfections, although poorly understood, are widely accepted to contribute to severe CAP. A clear picture of the etiology of CAP would help to estimate the potential impact of novel public health interventions, such as antiviral therapies in case management or influenza vaccination for prevention in vulnerable populations. In order to provide up-to-date data on the etiology of CAP, several studies were recently conducted, in particular two large studies in the USA in 2010–2012, targeting children and adults ^11, 12^, and two studies that targeted under-fives in low and middle income countries, the GABRIEL Pneumonia Study in 2010–2014, and the Pneumonia Etiology Research for Child Health (PERCH) study in 2011–2014 ^13^. These studies pointed to the importance of viruses in the etiology of CAP.

LRTI are a major cause of morbidity and mortality in the acute phase of humanitarian crises. Case management (diagnoses, treatment choice and delivery) and public health interventions (immunization strategies) for CAP in humanitarian crisis settings are conducted blindly or based on the assumption that evidence gathered in non-crisis settings applies to displaced populations. However, large knowledge gaps remain in many areas, including the etiology of these infections ^14, 15^. No studies have been specifically designed to assess the etiology of CAP in populations facing humanitarian crisis.

### Evaluation of the etiology of infection

The reference method for identifying the pathogen responsible for an infection is to sample the infected tissues, i.e., the lung, in patients with pneumonia, and screen for pathogenic agents by culture or molecular tools. Such invasive procedures are difficult to set up, even in hospital settings, and virtually impossible in primary health care. To circumvent this issue, the causative agent can be identified using samples from the upper respiratory tract using non-invasive techniques, e.g. nasopharyngeal swabs ^11, 12^. Due to the existence of healthy carriers of several potential pathogens (e.g. *Streptococcus pneumoniae*) in the population, there is a need to adjust for carriage: the higher the proportion of asymptomatic carriers of a given pathogen, the lesser the chances that this agent is the etiology of CAP when found in the upper respiratory tract.

In the last decade, transcriptomic analyses of the blood of patients infected with different pathogens have revealed gene expression patterns that correlate strongly with individual etiologic agents ^16, 17^. White blood cells express different genes in response to infection with different agents, and these patterns can be used to distinguish viral and bacterial infections with high accuracy ^18^. These transcriptomic patterns can even distinguish between different viral (or bacterial) infections, and also enable evaluation of the severity of infection. RNA sequencing analysis of the gene expression profiles of white blood cells during infection enables differentiation of viral and bacterial infections with higher specificity than white blood cell counts ^19^. Therefore, transcriptomics represents an innovative tool that reduces the need to identify pathogens by culturing respiratory tract samples and does not need to be adjusted for asymptomatic carriage. The ideal control samples for transcriptomic analysis are samples from healthy patients, i.e. those attending for vaccination or other routine healthcare visits.

### Situation of Syrian refugees in Lebanon

Lebanon is severely affected by a complex, chronic and protracted refugee crisis due to the ongoing war in Syria that began in March 2011. The Bekaa valley is the main entry point for Syrian refugees, followed by the North Lebanon region and Beirut, with refugees settling all over the country. The crisis not only affects Syrian refugees, but also the local Lebanese population and Palestinian refugees who settled decades ago. The majority of Syrian refugees reside in approximately 4000 informal tented settlements and mobile centers, garages and unfinished buildings, with no officially established access to food, water, sanitary means, health or education.

Respiratory tract infections (RTI) are among the leading causes of morbidity and mortality in children and adults affected by the current humanitarian crisis in Lebanon. According to Médecins Sans Frontières (MSF), RTI accounted for at least 56% of ambulatory or in-patient health care visits in the first six months of 2015, and up to 79% of visits among children under 5-years-old in the healthcare facilities attended mainly by refugees in the Bekaa valley (MSF, personal communication). Unpublished data from primary healthcare facilities in Lebanon suggests that 10–25% of these cases of RTI are CAP (Ali Ghassani, personal communication), though data on the etiology and severity of these cases of CAP cases is lacking.

In Lebanon, CAP peaks between October and April. Specifically, 80% of the primary consultations for children during winter are related to RTI, compared to 20% in the summer (Ali Ghassani, personal communication). The Lebanese surveillance program for pulmonary infections due to *S. pneumoniae* considers the general population, and therefore current data on the incidence, predominant serotypes, and antimicrobial susceptibility of CAP among the refugee population is not available ^20^. UNICEF and the Lebanese Ministry of Health started the introduction of a 13-valent pneumococcal vaccine (PCV) as part of a National Program in 2016, among children of Syrian, Palestinian and Lebanese origin.

### Objectives

In order to fill the knowledge gaps described above, the Pneumonia Etiology Among Refugees and the Lebanese population (PEARL) study was initiated in 2016 to identify the causal pathogenic microorganisms in the respiratory tract of children and adults presenting with signs of CAP to health facilities run by medical associations in the context of a humanitarian crisis. The first inclusions took place in November 2016, and the study is expected to last until March 2018.

The primary objective of the PEARL study is to estimate the population attributable fractions (PAFs) of specific viral and bacterial pathogens, i.e. the proportion of CAP attributable to each pathogen, in both the refugee population and Lebanese population. The goals behind this objective are (i) to enable local healthcare staff to provide more accurate diagnoses and improved case management and care, (ii) to help designing antimicrobial stewardship interventions, and (iii) to help assessing the impact of PCV, as it will generate baseline data on the burden of CAP caused by *Streptococcus pneumoniae* at the introduction phase of PCV in the national vaccination program.

The secondary objectives of the PEARL study are to: identify *S. pneumoniae* serotypes in nasopharyngeal and blood samples; identify the antimicrobial susceptibility profiles of the pathogenic bacteria isolated from nasopharyngeal and blood samples and compare these profiles with antibiotic prescriptions; examine the association between respiratory viral infections and invasive pneumococcal infections; identify risk factors for CAP in this population, especially those that may be modifiable (crowding, tobacco smoking, domestic sources of smoke produced by cooking or heating, etc.), and thus provide data for prevention programs; provide current data on the incidence and severity of CAP in vulnerable populations in Lebanon; provide a unique transcriptomics dataset as regards the sociodemographic profile of the patients and spectrum of diseases; compare microbiological and transcriptomic methods in estimating viral vs bacterial attributable fractions of LRTI; and assess the operational capacity of rapid, nuclear acid-based point-of-care diagnostic tests in a humanitarian crisis.

## Protocol

### Study setting and design

**Figure 1.**
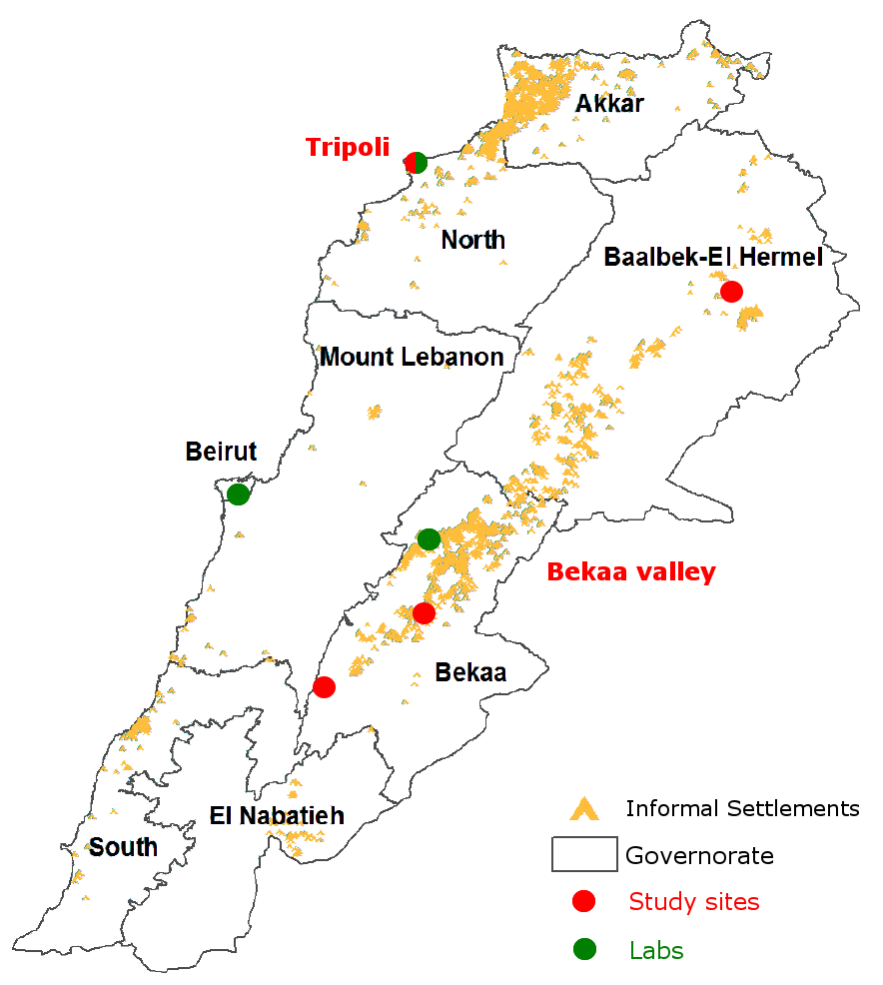
Concentration of Syrian refugees in Lebanon (source: UNHCR ^21^, image adapted under CC-BY 3.0 license) and location of the PEARL study sites.

Based on refugee density, two main regions were selected for this multicentric prospective case-control study: the Bekaa valley and the city of Tripoli ( Figure 1). Both regions have similar demographic composition, dynamics and climate. The population eligible for the study consists of any child (>2-months-old) or adult attending one of four primary healthcare facilities (no hospital facilities) that provide routine health care and immunization: one site located in Tripoli and three sites in the Bekaa valley (Kamed el Loz, Machghara, and Baalbeck El Ain). In addition to refugees, local and vulnerable Lebanese individuals who also attend these health centers (although to a lower extent) will be included for ethical and practical reasons.

Nasopharyngeal, urine and blood samples will be collected from patients with CAP (cases). Adults with CAP able to produce sputum will be asked to provide a sputum sample.

As CAP-related pathogens can also be carried by healthy individuals, control individuals will be recruited from the same health facilities; cases and controls will be matched in a 1:1 ratio by age, season and site. Nasopharyngeal swabs and urine will be collected from all controls; a subset of controls (15%) will also be asked to provide a small blood sample (500–1000 µL) for transcriptomic analysis. Controls will not be asked to provide sputum samples.

After enrolment in the study, patients will be managed as per local guidelines and doctors’ recommendations; in particular, the study won’t alter procedures for referral for complementary examinations or hospitalization.

### Inclusion criteria

***Cases.*** Physicians will evaluate case definitions on the basis of a clinical examination and patient history. The case definition was based on WHO’s IMCI ^22^. Cases should meet all of the following inclusion criteria: (i) patient aged > 2 months, (ii) with cough or dyspnea, (iii) lower chest wall indrawing (in children ≤ 3 years only) or tachypnea, (iv) no wheezing suggestive of asthma at auscultation, (v) onset of symptoms within the last 14 days, (vi) and informed consent statement signed by the patient, parent, or legal guardian ( Supplementary File 1). Tachypnea is defined as > 50 breaths per minute in patients between 2-months and < 1-year-old; > 40 breaths per minute, between 1 and < 5-years-old; 30 breaths per minute, between ≥ 5 and <18-years-old and > 20 breaths per minute, if ≥ 18 years-old. Characteristics of wheezing suggestive of asthma included: expiratory wheezing, high pitched wheezing, wheezing with history of asthma and without fever nor history of fever, and any clinical picture such as the clinician would exclude LRTI and retain asthma as final diagnostic.

Exclusion criteria are (i) any characteristic of healthcare-associated pneumonia, (including hospitalization at an acute care hospital for 2 or more days within 90 days of infection, residence in a nursing home or long-term care facility, recent intravenous antibiotic therapy, or wound care from medical staff within the 30 days prior to the current infection); or (ii) increased risk of lower airway disease, such as immunocompromised status due to underlying disease, including hemodialysis, or immunosuppressant treatment; or (iii) treatment with inhaled corticosteroids or other asthma medications.

The study nurse will check inclusion criteria, and ask the patient or their parent/guardian to provide signed informed consent and fill in the case report form (CRF), which includes a medical history (e.g. HIV infection, tuberculosis, respiratory infections), risk factors for pneumonia, prior medical treatment and immunizations, description of clinical signs that warrant enrolment in the study, current and recent treatment (antibiotic therapy), disease progression, and socio-economic characteristics ( Supplementary File 2). Pulse oximetry is used to assess the severity of CAP ^23^; pulse oximeters will be made available to all four healthcare facilities for the present study and pulse oximetry will be reported in the CRF. Data will be anonymized by attributing a unique patient identification code number issued at enrolment of each patient.

Physicians may request other tests such as chest radiography without interfering with the study protocol. Antimicrobial treatment may begin immediately after blood sampling, if required. Cases and controls may also undergo clinical and lab tests as required. Chest X-ray is not required as this examination is not available at all health care facilities, especially the three remote healthcare facilities in Bekaa valley; if available, results from chest X-rays will be reported in the CRF.

Severity of pneumonia will be classified according to the IMCI/WHO guidelines ^22^. Moreover, in children under five years of age, we will assess the breathing rate twice, since it has been shown that the cut-off of > 50 breaths per minute, assessed twice, is more specific for pneumonia than the standard IMCI recommendations for children 1–5 years old (40 breaths/min) ^24^, and was successfully adopted in the ALMANACH protocol ^25, 26^.

***Controls.*** An aged-matched control will be recruited for each case by age group (± 1 year for children aged 2 months to 4 years, ± 2 years for children 5–17 years, and ± 5 years for patients aged 18–49 years old and adults aged ≥ 50 years old). Controls will be matched to cases attending the same site in the same calendar month (some flexibility is allowed since controls for cases included at the end of one month may be recruited in the next calendar month). The study nurse will identify next patient consulting for reasons other than respiratory (upper or lower respiratory tract) or gastrointestinal infection, check eligibility, obtain written informed consent from the participant, and fill in the CRF.

Controls should meet all of the following inclusion criteria: (i) patients aged > 2 months attending one of the four sites participating in the study for symptomatic disease or immunization; and (ii) informed consent signed by patient or parent/guardian. Patients will be excluded as controls if they (i) exhibited any symptom of RTI (cough, dyspnea, chest wall indrawing, tachypnea, fever, coryzal symptoms/“cold”) or (ii) intestinal infection (watery/bloody diarrhea, abdominal cramps) in the last 5 days. Individuals with gastro-intestinal symptoms were excluded as some viruses (adenovirus, enterovirus, and coronavirus) are proven or suspected to cause either respiratory or gastro-intestinal symptoms ^27– 29^, and including such patients as controls would result in an underestimation of the attributable fraction of these pathogens.

### Sample size calculation

As pathogen distributions can vary according to patient age, the univariate and multivariate analyses will be stratified by age group. We calculated the total sample size required for each age group to detect a difference in pathogen prevalence between cases and controls (assuming an equal distribution of samples between the age groups) based on a power of 90% (α = 0.05). Pearson’s χ ^2^ test indicated 150 cases and 150 controls were required in each of the four age strata (2 months to 4 years, 5–17 years, 18–49 years, > 50 years) to detect a 15% difference in pathogen frequency between cases and controls (with a carriage prevalence < 10% in controls) or detect a difference in pathogen frequency of 20% (with a carriage prevalence of up to 30% in controls). If no differences between age groups is observed, pooling the data from age groups will increase statistical power.

Thus, the final sample size will be a minimum 600 cases and 600 controls with the aim of including a maximum of 900 cases and 900 controls.

### Analysis of biological samples

**Figure 2.**
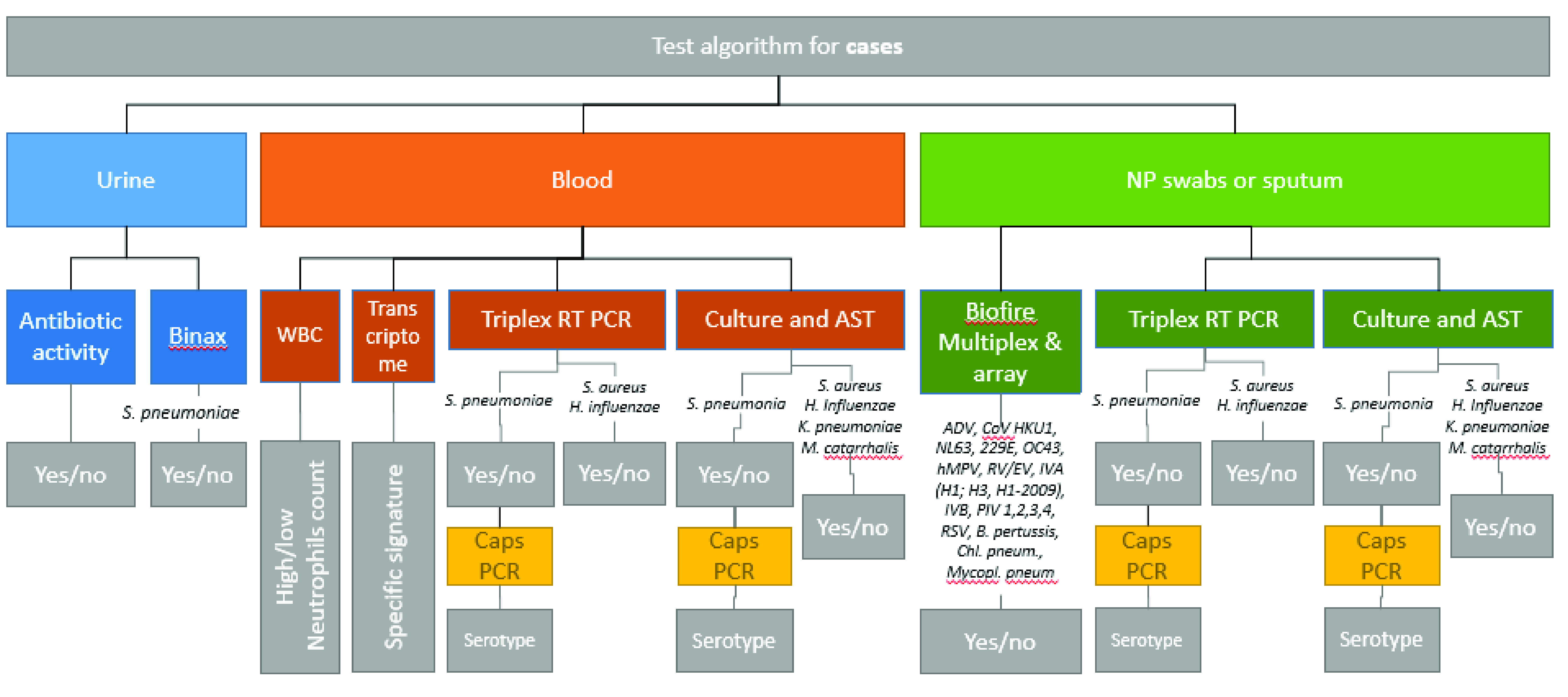
Sample analysis for cases. NP, nasopharyngeal; WBC, white blood cells; AST, Antimicrobial Susceptibility Testing; Caps PCR, capsular antigen serotyping PCR.

**Figure 3.**
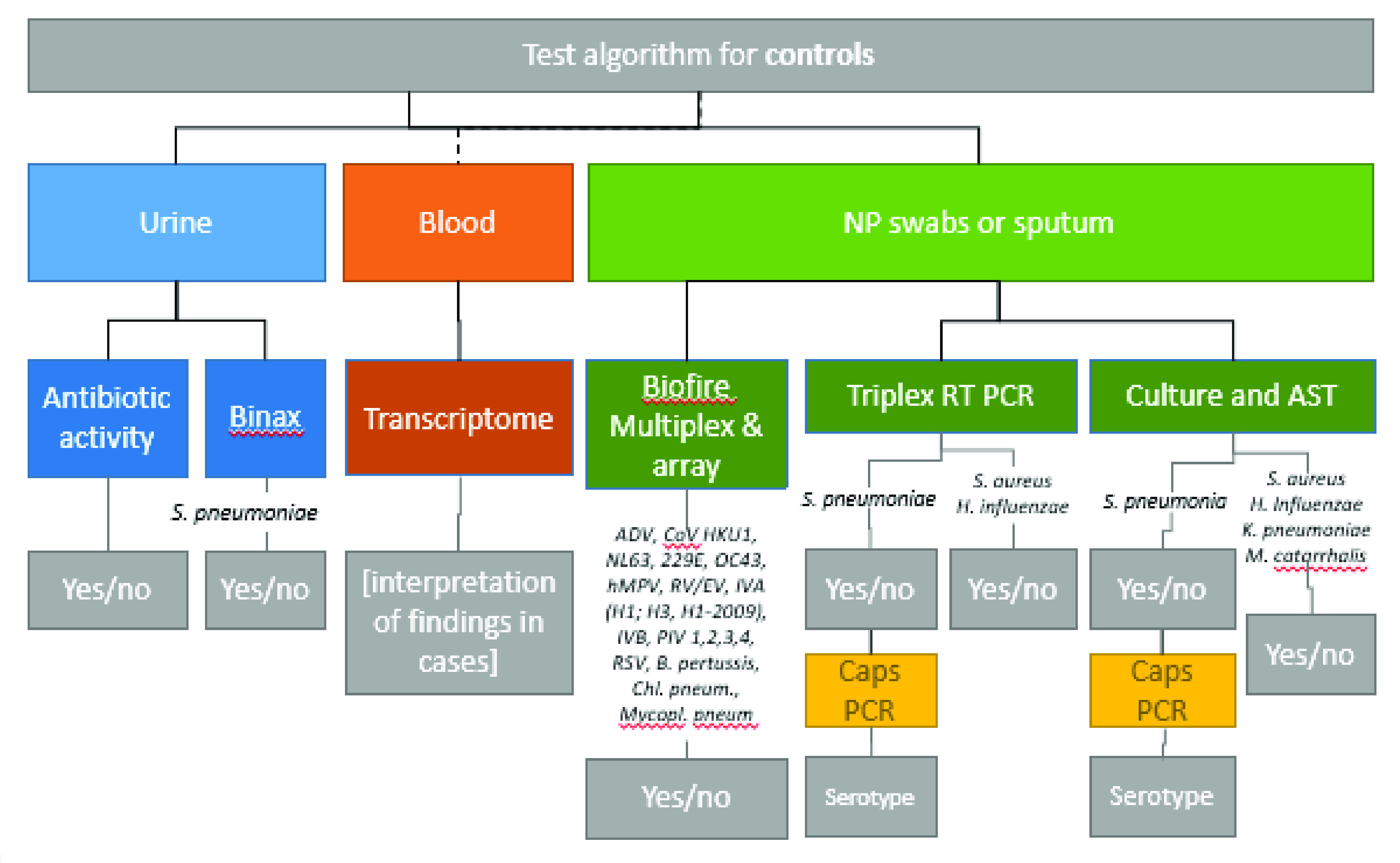
Sample analysis for controls. NP, nasopharyngeal; AST, Antimicrobial Susceptibility Testing; Caps PCR, capsular antigen serotyping PCR.

Biological samples will be collected and stored using standard protocols and transported together with the sample log to the laboratories in a multicentric manner ( Table 1). All analytical tests will be performed according to good clinical laboratory practice (GCLP) ^30^ following standard operating procedures defined for the study. Sample processing for cases and controls are illustrated in Figure 2 and Figure 3, respectively. The total volume of blood taken is 0.5-1.0 mL in controls of all ages and, in cases, 2.5-5.0 mL in infants (<1 year old), 3.5-6.0 mL in children aged one to four, 10.5-17.0 and 6 mL in children aged five or above, and 18.5-19.0 mL in adults (≥18 years old), with a flexibility in the age cut-off depending on the individual’s weight and overall health status.

**Table 1.** Collection, storage, processing and analysis of clinical samples.

Specimen	Collection container	Storage temperature	Assay	Processing	Laboratory
**Blood**	Blood culture bottle	+4°C	Blood culture and AST	Real time	CH, LMSE
	Tempus	+4°C/-20°C	Transcriptomics	Batch	NCH
	EDTA tube	+4°C	WBC	Real time	EB, CH
		+4°C/-80°C	Triplex PCR	Batch	LRM, LMSE
**Urine**	Sterile container (or sterile adhesive bags for babies)	+4°C	Antibiotic activity *	Batch	LRM, LMSE
		+4°C	Binax	Real time	CH, LMSE
**Nasopharyngeal swab**	Viral transport medium	+4°C/-80°C	Triplex PCR	Batch	LRM, LMSE
		+4°C/-80°C	Micro-array (BioFire) *	Real time	
		+4°C/-80°C	*S. pneumo* CAPS-PCR	Batch	
**Nasopharyngeal swab**	STGG medium	+4°C	Bacterial culture *	Real time	CH, LMSE
		+4°C/-80°C	CAPS-PCR	Batch	LRM, LMSE
**Sputum** (adults only)	Sterile container	+4°C	Bacterial culture	Real time	CH, LMSE
		+4°C/-80°C	Triplex PCR	Batch	LRM, LMSE
		+4°C/-80°C	Micro-array (BioFire) *	Real time	
		+4°C/-80°C	*S. pneumo* CAPS-PCR	Batch	

*: assay also performed in controls; CH: Chtoura hospital; LMSE: Laboratoire Microbiologie Santé et Environnement; LRM: Laboratoire Rodolphe Mérieux; NCH: Nationwide Children’s Hospital; EB: El Bashaer health center.

Clinically relevant results will be communicated to the clinicians in a timely manner. Such results include: (i) identification of pathogen bacteria in blood, by conventional microbiology or molecular tools, (ii) identification of clinically relevant bacteria in nasopharyngeal swab or sputum of cases, e.g. detection of atypical bacteria; (iii) identification of RSV in infants (cases or controls) aged ≤12 months, and (iv) positive Binax® test in urine of case. Whenever the lab identifies a pathogen or sensitivity profile of importance for the clinical management of pneumonia, they will provide their etiological diagnosis to the clinical team to ensure appropriate case management. All laboratory data will also be transmitted to the local Fondation Mérieux office in Beirut.

***Molecular biology testing of respiratory samples.*** A trained nurse or lab technician-in-charge will collect two nasopharyngeal samples from all cases and controls ^31^: one will be discharged in virus transport medium (VTM) and the other in skim milk-tryptone-glucose-glycerol (STGG) medium. Nasopharyngeal swabs will be processed and analyzed as shown in Table 1 and Figure 2 and Figure 3.

 Nucleic acids extracted from 350 μL of VTM using QIAamp® DNA mini kit (Qiagen, Hilden, Germany) will be subjected to a real-time triplex PCR assay targeting *Streptococcus pneumoniae, Staphylococcus aureus* and *Haemophilus influenzae B,* the three most common bacteria detected in CAP. This triplex PCR has been used in previous studies ^32, 33^. Briefly, 5 μL extracted nucleic acids are added to 9 μL Takyon No ROX Probe 2X MasterMix dTTP (Eurogentec, Seraing, Belgium) and 4 μL of a solution containing 1 μM of each primer and probe. Each PCR mixture is submitted to 95°C for 10 min then 40 cycles of 8 sec at 95°C then 34 sec at 60°C. The respective 5’-3’ sequences of forward primers, reverse primers and probes are: lytA gene (S. pneumoniae) ACG AAT AAC CAA CCA AAC AAC, CCA GTA GCC AGT GTC ATT C, and tca Atc Gtc Aag Ccg ttc t using HEX as a fluorophore (capital letter in probes indicate locked nucleic acid substitution); vicK gene (S. aureus) GAA GCA GTC TAA CCG TAG TC, GGG ATA TTA TAT ACC CAG ACA CG, and tcc Tta Cca Ccg Cca taa, using FAM as a fluorophore; bexA gene (H. influenzae B) ATT TGA GAA ACG CAA AGA CC, ATT TGA GAA ACG CAA AGA CC, and agt Ttc Aca Tag Ccc gag t, using Cy5 as a fluorophore. Amplification curves will be examined individually by two independent technicians, at the local laboratory and at the Laboratoire des Pathogènes Emergents (Lyon, France). Cycle thresholds (Ct) value will be manually set so that it intersects the exponential curve at its inflexion point. Any exponential signal observed between 0 and 40 Ct value will be considered as positive.

The FilmArray Respiratory Filmarray (BioFire Diagnostics, Salt Lake City, UT, USA) will be used to identify pathogens that cause pneumonia from VTM ^34^ and sputum samples. The 17 viruses and three atypical bacteria detected by this assay are adenovirus (ADV), coronavirus (CoV) HKU1, coronavirus NL63, coronavirus 229E, coronavirus OC43, hMPV, human rhinovirus (RV)/enterovirus (EV), influenza virus A (IVA), influenza A/H1, influenza A/H3, influenza A/H1-2009, influenza B, parainfluenza virus (PIV) 1, parainfluenza virus 2, parainfluenza virus 3, parainfluenza virus 4, RSV, *Bordetella pertussis, Chlamydophila pneumoniae,* and *Mycoplasma pneumoniae.*

Samples from cases positive for *Streptococcus pneumoniae* by conventional microbiology or triplex PCR will be serotyped using a Multiplex Real-Time PCR assay ^35^ that can identify 40 capsular antigen serotypes (1, 2, 3, 4, 5, 6A/B, 6C, 7C, 7F, 8, 9N/L, 9V, 10A, 10F, 11A, 12F, 13, 14, 15A, 15B/C, 16F, 17F, 18C, 19A, 19F, 20, 21, 22F, 23A, 23B, 23F, 24, 31, 33F, 34, 35A, 35B, 35F, 38, 39) and includes an internal control ( *LytA*).

***Culture of bacteria from respiratory samples.*** Sputum samples and STGG media will be inoculated onto different selective agar plates according to standard laboratory procedures (at least one blood culture medium, one chocolate agar, and one medium selective for gram-negative bacilli) and incubated under specific conditions (37°C, aerobic atmosphere supplemented with CO _2_) to determine the presence of respiratory pathogens and assess antibiotic susceptibility ^36^. Quality of sputum and absence of contamination by saliva will be assessed by standard laboratory procedures ^36^. Positive cultures will be subjected to Gram staining and examined by light microscopy, and sub-cultured for identification and antimicrobial susceptibility testing if the strain is confirmed to be clinically relevant.

***Detection of bacteria in blood.*** Duplicate aerobic haemoculture assays will be performed for all cases using BacT/ALERT (bioMérieux, Marcy l'Etoile, France) or BACTEC (Becton Dickinson, Franklin Lakes, NJ, USA) automated blood culture systems. The target blood volume for blood culture is 1-3 mL in under-fives and 6 mL in children aged five or above and in adults, following manufacturers’ recommendations. If the volume of blood recovered is insufficient to inoculate two bottles, a single culture will be performed. Positive cultures will be examined by light microscopy (Gram staining) and subcultured on agar culture media for identification. Antimicrobial susceptibility testing will be performed for all clinically relevant strains.

Whole blood (200 μL EDTA) samples from all cases will be extracted using QIAamp DNA blood mini kit (Qiagen, Hilden, Germany), and the same semi-quantitative multiplex Real-Time PCR assay as for respiratory samples will be conducted on 5 μL of extracted DNA to identify *Streptococcus pneumoniae, Staphylococcus aureus* and *Haemophilus influenzae B*.

***Transcriptomics.*** Blood samples (0.5–1 mL) for transcriptomics from all cases and 15% of controls will be collected in Tempus tubes containing stabilizing agents for conservation of mRNA, and shipped to the Nationwide Children’s Hospital Research Institute, in Columbus, Ohio, USA. Sampling for transcriptomics was initiated in May 2017.

Total RNA will be isolated from whole blood collected in Tempus tube and analyzed for quality using the RNA 6000 Nano Kit (Agilent Technologies). Globin mRNAs will be further removed with GOBINclean kit (Thermo Fisher Scientific). Poly(A)-enriched next-generation sequencing library construction will be performed using the KAPA mRNA Hyper Prep Kit (KAPA Biosystems) with 500 ng of input total RNA and 11 amplification cycles according to the manufacturer’s protocol. Individual libraries will be quantitated via quantitative PCR using the KAPA Library Quantification Kit, Universal (KAPA Biosystems) and equimolar pooled. Final pooled libraries will be sequenced on an Illumina HiSeq 2500 with single-end 70-base-pair (bp) read lengths.

***Urinalysis.*** The presence of antibiotics in urine samples from all cases and controls will be assessed using the disk diffusion method ^37^, and the rapid immunochromatographic Binax® assay (Alere, Orlando, FL, USA) will be used for qualitative detection of *S. pneumoniae* C polysaccharide antigen in urine ^38^.

### Other data collected

All involved healthcare facilities will be required to provide monthly report of the total number of consultations and, whenever available, the number of LRTIs. This will help weighting the monthly results from the study to reflect the actual incidence of CAP. A subgroup of patients or parents/legal guardians, randomly selected, was called back at least one month after inclusion to re-evaluate the outcome of included cases (hospitalization, death).

### Study outcomes

The primary outcome of the PEARL study is the etiological distribution of CAP, expressed as PAFs for individual pathogenic agents. PAFs will be calculated for each three-month period of the 15-month study, and by patient age group (2 to 11-months-old and 1 to 4, 5 to 17, 18 to 49, and ≥ 50-years-old), by site location (Tripoli vs. Bekaa valley), subpopulation (Syrian refugees versus Lebanese population), and severity of pneumonia.

Additional indicators will be examined to assist data interpretation and for public health and clinical care purposes, including the incidence of RTI and other diseases in the populations consulting the four healthcare facilities involved in the study, agent-specific hospitalization and fatality rates, the socio-demographic characteristics of the patients, clinical signs of CAP (e.g. severity criteria), epidemic features if appropriate (e.g. types of influenza viruses circulating during epidemic periods), and care provided (e.g. antimicrobial use).

### Data management and analysis

All data (CRF, informed consent forms, data logs) will be transmitted to the local Fondation Mérieux office in Beirut for data entry using EpiInfo version 7.2 (CDC, Atlanta, GA, USA). The data will be anonymized by attributing a unique patient identification code number issued at patient enrolment. Access to the names of participants and their corresponding identification codes will be restricted and forms will be secured in locked cupboards.

Data analyses will include (i) descriptive comparisons of the sociodemographic, clinical, and lab data of cases and controls; (ii) univariate/multivariate logistic regression of the relationship between case/control status and pathogens, adjusted for age, season, site, and pneumonia risk factors; and (iii) computation of PAFs for every pathogen, specified by age group, period, study site, subpopulation (Syrian refugees, or general Lebanese population), and pneumonia severity, as described previously ^39^. Ninety-five-percent confidence intervals will be calculated, with regards to the outcomes linked to the primary and secondary objectives. Analyses will be performed using R version 3.3.2 (R Core Team, Vienna, Austria ^40^) and/or Stata version 13.0 (StataCorp LP, College Station, TX, USA) software. RNA sequencing data from transcriptomics will be analyzed at the Nationwide Children’s Hospital, OH, USA. Quality control of raw reads will be performed with FASTQC. Reads will be aligned to the reference human genome (GRCh38) using hisat2 after quality and adapter trimming by cutadapt. FeatureCounts program will be used to quantify total number of read counts for each gene. The RNA sequencing data analysis will be performed in the R programming language, using DESeq2 R package for size factor, dispersion estimation calculation and differential gene expression analysis. A number of supervised and unsupervised analytical approaches, and modular analyses will be used to identify the pathogen-associated transcriptome profiles as previously described ^41– 43^. Etiologic fractions will be also computed from transcriptomics data, independently from calculations from microbiological data, and compared with each other.

### Dissemination of the study outcomes

The results of the study (microbiological and transcriptomics results) will be published as scientific publications in international peer-reviewed journals. The designation of co-authors will conform to international guidelines governing publications.

The findings of the project, when completed, will be presented to the Ministry of Health of Lebanon, and to the medical and scientific community, such as United Nations agencies and non-governmental organizations, involved in medical care of vulnerable populations in Lebanon. The results will also be communicated via the GABRIEL website ( https://www.gabriel-network.org/). The study data relevant to a publication authored by the investigators will be available for review in a public data repository

## Ethical considerations

This study will be conducted in accordance with the Declaration of Helsinki ^44^, the recommendations for Good Ethical Practices in Epidemiology of the Association of French-language Epidemiologists ^45^, the Good Clinical Practice recommendations from the International Conference on Harmonization of Technical Requirements for Registration of Pharmaceuticals for Human Use (ICH) relevant to observational studies ^46^, and the GCLP guidelines of the WHO ^30^. The informed consent statement has been translated into Arabic and was validated during the pilot phase of the study. All adult patients and the parents or legal guardians of children (i.e. ≤18 years) will be asked to sign an informed consent statement prior to enrolment in the study.

As no national ethic committee exists in Lebanon, the study protocol and all other documents related to the trial (informed consent, CRFs, amendments) have been approved by the institutional review boards of the following organizations: El Bashaer Association, Tripoli, Lebanon (5 ^th^ December 2015); Université Libanaise, Ecole doctorale des Sciences et Technologies, Tripoli, Lebanon (3 ^rd^ December 2015); Amel Association, Beirut, Lebanon (9 ^th^ December 2015); and Université Saint-Joseph, Beirut, Lebanon (10 ^th^ November 2016).

## Current status of the study

Patient enrollment began in November 2016 and was completed in March 2018. At the time of submission of the first version of the manuscript, data cleaning and analysis was ongoing.

## Discussion/conclusions

The Pneumonia Etiology Among Refugees and Lebanese population (PEARL) study will yield unique data on the etiology of CAP in the context of a humanitarian crisis. This study is not only original in terms of target population (refugees), but also as it will assess patients of all ages and adopt a primary health care approach while previous major studies only targeted hospitalized cases ^2– 5, 7, 13^. Therefore, this study may provide a unique and valuable perspective on the etiology of CAP in the context of a humanitarian crisis.

However, assessment of the etiology of CAP in low resource primary health care settings relies on clinical signs for case definition, as chest X-rays are not available at most of the centers in this study. Inclusion criteria for cases of pneumonia are a controversial issue. Several case definitions, based on different clinical, radiological, or biological criteria, have been used in different studies. Opting for a single case definition imposes restrictions on the anatomical and histological levels of the respiratory tract involved, on the severity of disease, and therefore may introduce a bias towards the relative prevalence or contribution of specific pathogens. This makes it difficult to compare results between studies using different case definitions; the present study does not avoid this limitation.

We also anticipate other limitations. For example, the imperfect sensitivity of the diagnostic tests used to identify certain pathogens may result in an underestimation of certain etiological fractions ^47^. Moreover, with regards to surveillance of pneumonia, given that the population covered by the healthcare facilities participating in this study is hard to define, estimation of the precise incidence of CAP in this population might not be possible. Additionally, certain aspects of CAP will not be explored in this study, especially AIDS-associated pneumonia and tuberculosis. We expect individuals with HIV will be excluded from the present study as HIV infection is one type of immunosuppression, which is an exclusion criterion. As the prevalence of HIV is very low (< 0.1%) in both the general Lebanese and Syrian populations ^48^, inclusion of cases HIV/AIDS-associated CAP would only have a small effect on the results. Patients with tuberculosis are also expected to be excluded, since duration of symptoms >14 days is an exclusion criterion. The incidence rate of tuberculosis among Syrian refugees did not increase in the last couple of years and remains relatively low at close to pre-war levels, i.e. 10-20/100.000 population ^49^. Therefore, we consider it unlikely that tuberculosis is a major cause of acute LRTI in our population ^50^.

Finally, the external validity of our study is limited in time (15 months) and space (four health facilities). A case-control study cannot replace longitudinal surveillance systems, as are currently being set up in Lebanon, e.g. the Lebanese Inter-Hospital Pneumococcal Surveillance Program ^20^. Therefore, although the situation of Syrian refugees in Lebanon shares a lot of characteristics with other crises, one should be cautious about applying the results of PEARL to other humanitarian crises, because of the variations of the epidemiology of LRTI.

Despite these limitations, the PEARL study is expected to provide healthcare planners with an empirical basis for the management of CAP in the context of a refugee crisis. Such information may help to guide population-based health interventions, such as immunization strategies for pneumococci, *H. influenzae* and influenza. In particular, analysis of *S. pneumoniae* serotypes in vaccinated and non-vaccinated individuals will provide information on the herd effect following introduction of the PCV in Lebanon and will provide proxy baseline data for evaluation of the success of this vaccination program. This study will also provide practical experience and a methodology for determination of the etiology and involvement of viral and bacterial agents in CAP in other similar humanitarian crisis settings.

## Data availability

No data is associated with this article.

## Supplementary material

Supplementary File 1: Informed consent form.

Supplementary File 2: Case report form.
