# Supplementary material for: Investigating Pneumonia Etiology Among Refugees and the Lebanese population (PEARL): A study protocol
Source: Gates Open Res. 2019 Jun 13;2:19. Originally published 2018 Apr 18. [Version 2] doi: 10.12688/gatesopenres.12811.2 (PMC7569241; doi:10.12688/gatesopenres.12811.2)
Supplement: Supplementary file 1 [file gatesopenres-2-14036-s0000.tgz › 10f9b0d4-229e-4228-810e-3ec29945b181.pdf]

PEARL study - Informed Consent Statement (English)

Dr. ___________________________________, physician at _____*[name of the institution]*_________, presented the study on the surveillance of pneumonia currently underway in this health center.

You are invited to participate in the pneumonia research study named PEARL (Pneumonias' Etiology Among Refugees and Lebanese population) conducted in this health facility. The purpose of this study is to know the agents responsible for pneumonia among people who go to this clinic, including many Syrian refugees. The study has been approved by the Institutional Review Board of ________*[name of the institution]*_______________.

Before recruitment, you will be asked questions on medical history. We may also contact you further if we require more information.

As part of the research, urine and respiratory samples (two nasal swabs, and a sputum if you can) will be collected and sent to the study participating laboratories for processing. If you or your child presents pneumonia symptoms, blood samples will be also collected for pneumonia diagnostic purposes. If you agree, a small blood sample may be taken even if you have no pneumonia.

RISKS

Overall there are no risks involved in the sampling and study procedure. Nasal sampling is unpleasant since a swab must be inserted in the back of the nose, but not painful. Blood testing requires that a needle must be inserted in the vein, which can be a little painful.

BENEFITS

The testing will enable a better diagnosis and treatment of the disease as tests are an important indicator of the agent causing your illness. The cost of the test won't be charged to you and results will be made available to the practitioner who took care of you, if the results may influence your treatment. You can have access to these results, on your request. By participating you are also contributing to the better understanding of pneumonia causing agents to enable better treatment of this disease in your community.

COSTS

You do not have to pay or incur any extra expenditure for participating in this study.

CONFIDENTIALITY

Any information obtained about you in the course of the study, including your identity constitutes confidential information. This information will be used solely for the purposes of this study; no information will be revealed; the study will ensure that your information will remain confidential. A research number will be assigned to you in this clinic, and your name will not be used, nor released or shared with any third parties not involved in research. When the result of the study is published your name will remain confidential.

I am satisfied with the explanations written and oral that were provided and I was given the possibility to ask questions. If I have other questions during the course of the study, I can consult _______*[name of clinical investigator]*________clinical investigator, at the ________*[name of the institution]*________.

I have fully understood the aims of this study and the different steps composing it.

I have fully understood that I am free to agree or refuse to participate in this study and that I am free to withdraw from this study at any time.

I agree that my personal data, under the cover of anonymity (name absent, only code number provided) may be transferred to France for analysis. I have the right to access and to correct my personal data and I may exercise this right with the intervention of the clinical investigator.

I have understood that some of the biological samples for this study will be examined in laboratories in Chtoura hospital and Beirut / El Bashaer and Lebanese University in Tripoli, and Nationwide Children's Hospital in Columbus, Ohio (USA).

I have been informed that my samples will be stored and may be used at a later date for research on pneumonia, unless I am in disagreement with this.

Last name and first name of patient:……………………………………………………………………….

Phone numbers that can be used to contact the patient:

+961 I__I__|__|__I__|__I__|__|__|__| Name of phone owner:………………………………………….

+961 I__I__|__|__I__|__I__|__|__|__| Name of phone owner:………………………………………….

+961 I__I__|__|__I__|__I__|__|__|__| Name of phone owner:………………………………………….

Last name and first name of signatory:

....

Patient himself (if major) ☐ Mother ☐ Father ☐ Legal Guardian ☐

Date: __ __|__ __|__ __ __ __

Signature or, if the signatory is unable to write, left index fingerprint:

Last name and first name of investigator completing the consent statement:

………..

Date: __ __|__ __|__ __ __ __

Signature:

Study Subject coding:

استمارة الموافقة المسبق

قدم الدكتور______________________، الطبيب في ___________________________، دراسة عن مراقبة الالتهاب الرئوي التي يتمّ إجراؤها حاليًّا في هذا المركز الصحي.

أنت مدعو للمشاركة في دراسة بحثية حول الالتهاب الرئوي – Pearl – (مسببات الالتهابات الرئوية بين اللاجئين والسكان اللبنانيين) التي أجريت في هذا المركز الصحي. يكمن هدف هذه الدراسة في تحديد العوامل المسبّبة للالتهاب الرئوي بين الناس الذين يقصدون هذه العيادة، بما في ذلك العديد من اللاجئين السوريين. وقد وافق مجلس المراجعة المؤسسية في ____________________________ على هذه الدراسة.

قبل عملية التوظيف، ستُطرح عليك أسئلة حول تاريخك الطبي. وقد نتصل بك مرة أخرى إذا احتجنا إلى المزيد من المعلومات.

سيتم جمع عيّنات من البول والجهاز التنفسي (عيّنتان طبيتان وبلغم إذا كان ممكنًا) كجزء من البحث وسيتم إرسالها إلى المختبرات المشاركة بالدراسة من أجل معالجتها. إذا ظهرت لديك أو لدى طفلك أعراض الالتهاب الرئوي، سيتم جمع عينات من الدم أيضًا من أجل تشخيص الالتهاب الرئوي.

المخاطر

بشكل عامّ، ما من مخاطر مرتبطة بأخذ العينات وبإجراءات الدراسة. ويُعتبر أخذ العينات من الأنف أمرًا كريهًا، بما أنّه يجب إدخال ماسحة قطن في الجزء الخلفي من الأنف، ولكنّ ذلك ليس أمرًا مؤلمًا. ويتطلّب فحص الدم إدخال إبرة في الوريد، الأمر الذي يمكن أن يكون مؤلمًا قليلاً.

الفوائد

سيمنح الاختبار تشخيصًا وعلاجًا أفضل لهذا المرض، إذ إنّالاختبارات تعتبر مؤشرًا هامًا للعامل الذي يسبّب مرضك. لن تتحمّل تكاليف الاختبار وستكون النتائج متاحة للطبيب الذي اهتمّ بك، في حال كانت النتائج قد تؤثر على علاجك. يمكنك الحصول على هذه النتائج، بناء على طلبك. ومن خلال المشاركة، أنت تساهم أيضًا في فهم العوامل المسببة للالتهاب الرئوي بشكل أفضل من أجل تعزيز علاج أفضل لهذا المرض في مجتمعك.

التكاليف

لا يجب أن تدفع أو تتحمل أي نفقات إضافية من أجل الإشتراك في هذه الدراسة.

السرية

ستبقى أي معلومة تم الحصول عليها عنك في سياق هذه الدراسة، بما في ذلك هويتك، سريّة. وسيتم استخدام هذه المعلومات فقط لتلبية أهداف هذه الدراسة؛ لن يتم الكشف عن أي معلومة. وستضمن الدراسة أن تبقى المعلومات الخاصة بك سرية. وسيتمّ إعطائك رقم خاص بالدراسة في هذه العيادة، ولن يتم إستخدام اسمك، أو مشاركته مع أي طرف ثالث لم يشارك في البحث. وعندما يتمّ نشر نتائج الدراسة سيبقى اسمك سريًّا.

أنا راض عن التفسيرات المكتوبة والشفوية التي تمّ تقديمها وقد تمّ منحي الفرصة لطرح الأسئلة. وإذا كان لدي أسئلة أخرى أثناء الدراسة، فيمكنني استشارة المحقق العيادي ______________________، في ______________________.

لقد فهمت تمامًا أهداف هذه الدراسة والخطوات المختلفة التي تكوّنها.

لقد فهمت تماما أنني حرٌّ في أن أوافق أو أرفض المشاركة في هذه الدراسة، وأنني حرٌّ في أن أنسحب من هذه الدراسة في أي وقت.

أوافق على أنه قد يتم نقل بياناتي الشخصية إلى فرنسا للتحليل، تحت غطاء إخفاء الإسم (الاسم غير مذكور، يتم فقط تزويد رقم الرمز). ولدي الحق في الحصول على بياناتي الشخصية وفي تصحيحها وقد أمارس هذا الحق مع تدخل المحقق العيادي.

لقد فهمت أنه قد يتم فحص بعض من العينات البيولوجية الخاصة بهذه الدراسة في مستشفى شتورة و جامعة سان جوزف في بيروت / جمعية البشائر و الجامعة اللبنانية في طرابلس /مستشفى الأطفال على الصعيد الوطني في كولومبوس (أوهايو، الولايات المتحدة الأمريكية)

لقد تمّ إبلاغي أنه سيتم تخزين العينات الخاصة بي وأنّه قد يتم استخدامها في وقت لاحق في دراسة حول الالتهاب الرئوي، إلا إن لم أوافق على ذلك.

اسم المريض الكامل: ....

أرقام الهواتف التي يمكن استخدامها من أجل الاتصال بالمريض:

ا__ | __ا__ | __ا__ا__ | __ | __ | __ |961+ اسم صاحب الهاتف...............................

ا__ | __ا__ | __ا__ا__ | __ | __ | __ |961+ اسم صاحب الهاتف...............................

ا__ | __ا__ | __ا__ا__ | __ | __ | __ |961+ اسم صاحب الهاتف...............................

الاسم الكامل للموقع : ....

المريض الرئيسي إذا كان قد بلغ سن الرشد ☐ الوالدة ☐ الوالد ☐ الوصي القانوني ☐

التاريخ: __ __ | __ __ | __ __ __ __ **التوقيع:** (إذا كان المريض أو الوالد أو الوالدة أو الوصي القانوني غير قادر على الكتابة: بصمة إصبع سبابة الأيسر)

الاسم الكامل للشخص الذي يملأ استمارة الموافقة المسبقة:

....

التاريخ: __ __ | __ __ | __ __ __ __ التوقيع:

ترميز موضوع الدراسة:

