# Supplementary material for: Investigating Pneumonia Etiology Among Refugees and the Lebanese population (PEARL): A study protocol
Source: Gates Open Res. 2019 Jun 13;2:19. Originally published 2018 Apr 18. [Version 2] doi: 10.12688/gatesopenres.12811.2 (PMC7569241; doi:10.12688/gatesopenres.12811.2)
Supplement: Supplementary file 2 [file gatesopenres-2-14036-s0001.tgz › d02e7add-0bac-4543-aa0e-4acb575e8c6c.pdf]

PATIENT'S STUDY IDENTIFICATION | | | | | | |

1. GENERAL DATA

101	DATE OF VISIT<br>*FORMAT DD/MM/YYYY, UNKN: 99/99/9999*	☐☐ / ☐☐ / ☐☐☐☐
102	NAME OF STUDY INVESTIGATOR<br>*PERSON FILLING THIS FORM*	. . . .
103	NAME OF CONSULTING PHYSICIAN	. . . .
104	LOCAL PATIENT ID<br>*(IF ANY)*	. . . .
105	ENROLLED AS CASE OR CONTROL?	CASE .... 1<br>CONTROL .... 2<br>CASE WITH TEMPUS .... 3<br>CONTROL WITH BLOOD SAMPLING (TEMPUS) 4

2. MEDICAL ASSESSMENT BY THE MEDICAL DOCTOR

201	RESPIRATORY RATE	☐☐ CYCLES PER MINUTE<br>*NOT REPORTED: 99*
202	RESPIRATORY RATE *(2ND ASSESSMENT IN CHILDREN <5Y)*	☐☐ CYCLES PER MINUTE<br>*NOT REPORTED: 99*
203	IS THE PATIENT AFFECTED BY A CHRONIC DISEASE ?<br><br>*CHECK ALL THAT APPLY*<br><br>*CAN BE FILLED ON THE BASIS OF PATIENT FILE, IF AVAILABLE AND COMPLETE*	HEART DISEASE ________________ A<br>*(DETAIL)*<br>DIABETES .... B<br>HYPERTENSION .... C<br>RENAL IMPAIRMENT .... D<br>LIVER DISEASE .... E<br>ASTHMA .... F<br>TUBERCULOSIS .... G<br>HIV/AIDS .... H<br>RHEUMATOLOGIC DISEASE .... I<br>CHRONIC NEUROLOGICAL DISEASE .... J<br>STROKE, HEMIPLEGIA, PARAPLEGIA .... K<br>CANCER, LEUKEMIA, LYMPHOMA .... L<br>COPD .... M<br>OTHER ________________ X<br>*(DETAIL)*<br>**NO CHRONIC CONDITION** .... Y<br>DON'T KNOW .... Z
204	IS THE PATIENT TREATED WITH IMMUNOSUPPRESSANTS?<br><br>*CAN BE FILLED ON THE BASIS OF PATIENT FILE, IF AVAILABLE AND COMPLETE*	NO .... 1<br>YES, INHALED CORTICOSTEROIDS .... 2<br>YES, ORAL CORTICOSTEROIDS .... 3<br>YES, CHEMOTHERAPY .... 4<br>YES, OTHER ________________ 8<br>*(DETAIL)*<br>DON'T KNOW 9

FOR CASES CONTINUE TO 205 - FOR CONTROLS, SKIP AND GO TO 301

205	DOES THE PATIENT PRESENT THE FOLLOWING SIGNS AT CHEST EXAMINATION?	LOWER CHEST WALL INDRAWING	A	
		CHEST PAIN	B	
		DULLNESS TO PERCUSSION	C	
		DIMISHED BREATH SOUNDS	D	
		RASPING, PLEURAL RUBS	E	
		FINE, HIGH PITCHED CRACKLES/RALES	F	
		COARSE, LOW PITCHED CRACKLES/RALES	G	
		RONCHI	H	
		LOW PITCHED WHEEZES	I	
		EXPIRATORY, HIGH PITCHED WHEEZES	J	
		OTHER ____________ *(DETAIL)*	X	
	CHECK ALL THAT APPLY	NONE OF THOSE SIGNS	Y	
		DON'T KNOW	Z	

			YES	NO	*(NOT ASSESSED)*
206	DOES THE PATIENT PRESENT, OR COMPLAIN OF THE FOLLOWING SYMPTOMS?	11 UPPER RESPIRATORY TRACT INFECTION (CORYZAL SYMPTOMS, RHINORRHOEA, RHINOPHARYNGITIS, SORE THROAT, OTITIS, CONJUNCTIVITIS)	1	2	9
		12 SKIN RASH	1	2	9
		13 LYMPADENOPATHY	1	2	9
		14 MYALGIA, ARTHRALGIA	1	2	9
		21 LETHARGY OR UNCONSCIOUSNESS	1	2	9
		22 CONVULSIONS	1	2	9
	PLEASE ANSWER ALL QUESTIONS	23 INABILITY TO DRINK	1	2	9
		24 PERSISTANT VOMITING	1	2	9
		25 STRIDOR IN A CALM CHILD	1	2	9
		26 SEVERE MALNUTRITION	1	2	9

207	HAS THE PATIENT BEEN PRESCRIBED **ANTIBIOTICS?**	YES	1	
		NO	2 →	GO TO 301
		DON'T KNOW	9 →	GO TO 301
208	WHAT ANTIBIOTIC HAS THE PATIENT BEEN PRESCRIBED?	AMOXICILLIN	A	
		AMOXICILLIN + CLAVULANIC ACID (AUGMENTIN)	B	
		CEFIXIME	C	
		CEFUROXIME	D	
		CEFTRIAXONE	E	
		LEVOFLOXACIN	F	
		CIPROFLOXACIN	G	
	CHECK ALL THAT APPLY	AZITHROMYCIN	H	
		CLARITHROMYCIN	I	
		COTRIMOXAZOLE	J	
		METRONIDAZOLE +/- SPIRAMYCIN	K	
		OTHER ____________ *(DETAIL)*	X	
		NAME UNKNOW	Z	
	UNKNOWN: 9999	DOSAGE [ ][ ][ ][ ]	MG/DAY	
	UNKNOWN: 99	DURATION [ ][ ]	DAYS	

3. CLINICAL HISTORY

301	PATIENT'S AGE	YEARS ..... [ ][ ] IF <1 YEAR OLD MONTHS .... [ ][ ]
302	DATE OF BIRTH *FORMAT DD/MM/YYYY, UNKN: 99/99/9999*	[ ][ ] / [ ][ ] / [ ][ ][ ][ ]
303	SEX	MALE .... 1 → GO TO 305<br>FEMALE 2

FOR ADULT PATIENTS, CONTINUE TO 304 - FOR CHILDREN, SKIP AND GO TO 307

304	Are you pregnant? *FOR WOMEN OF CHILDBEARING AGE ONLY*	YES .... 1<br>NO .... 2<br>NOT APPLICABLE .... 99<br>DON'T KNOW / DON'T WANT TO SAY 9
305	Are you a smoker?	YES .... 1<br>NO .... 2 → GO TO 311<br>DON'T KNOW / DON'T WANT TO SAY .... 9 → GO TO 311
306	How many cigarettes/shisha? *NONE: 00* *DON'T KNOW: 99*	[ ][ ] CIGARETTES PER [ ] DAY [ ] WEEK<br>[ ] SHISHAS PER [ ] DAY [ ] WEEK

FOR INFANTS (<1 Y OLD), CONTINUE TO 307 - FOR OLDER CHILDREN AND ADULTS, SKIP AND GO TO 311

307	Was the baby preterm? *PRETERM = LESS THAN 37 WEEKS GA*	YES .... 1<br>NO .... 2<br>DON'T KNOW 9
308	How much did the baby weight at birth?	[ ] . [ ] KG *UNKN: 9.9*
309	Is the baby exclusively breastfed?	YES .... 1<br>NO .... 2<br>DON'T KNOW 9
310	IS THE DEVELOPMENT APPROPRIATE FOR AGE? *(CLINICAL EVALUATION)*	YES .... 1<br>NO .... 2<br>DON'T KNOW 9

FOR CONTROLS CONTINUE TO 311 - FOR CASES, SKIP AND GO TO 315

311	What was the main reason why you came to the health centre? *CHECK ONLY ONE OPTION*	RESPIRATORY DISEASE .... 11<br>GASTROINTESTINAL DISEASE .... 12<br>FEVERISH DISEASE .... 13<br>SKIN DISEASE .... 14<br>BACK OR JOINT DISEASE .... 15<br>ACCIDENT, EXTERNAL INJURY .... 16<br>IMMUNIZATION .... 21<br>PREGNANCY (ANTENATAL VISIT) .... 22<br>FOLLOW-UP OF CHRONIC DISEASE (DIABETES, HYPERTENSION, ARYTHMIA, RENAL IMPAIRMENT,...) 23<br>OTHER ______________ *(DETAIL)* 80<br>ACCOMPANYING FAMILY MEMBER .... 91<br>DON'T KNOW / DON'T WANT TO SAY 99
312	Have you been hospitalized* last 3 months? **in an acute care hospital for ≥2 days within 90 days*	YES .... 1<br>NO .... 2<br>DON'T KNOW 9
313	Have you received medical wound care last month? *WITHIN 30 DAYS*	YES .... 1<br>NO .... 2<br>DON'T KNOW 9
314	Do you live in a nursing home or long-term care facility?	YES .... 1<br>NO .... 2<br>DON'T KNOW 9

315	Does someone in your house smoke?	YES	1
		NO	2
		DON'T KNOW	9
316	Do you currently feel fever or did you have fever last 2 weeks?	YES	1
		NO	2
		DON'T KNOW	9
317	Did you take antibiotics last month?	YES	1
		NO	2 → GO TO 320
		NOT APPLICABLE	3 → GO TO 320
		DON'T KNOW	9 → GO TO 320
318	What antibiotic did you take?	AMOXICILLIN	A
		AMOXICILLIN + CLAVULANIC ACID (AUGMENTIN)	B
		CEFIXIME	C
		CEFUROXIME	D
		CEFTRIAXONE	E
		LEVOFLOXACIN	F
		CIPROFLOXACIN	G
		AZITHROMYCIN	H
		CLARITHROMYCIN	I
		COTRIMOXAZOLE	J
		METRONIDAZOLE +/- SPIRAMYCIN	K
		OTHER ______________________	X
	CHECK ALL THAT APPLY	*(DETAIL)*	
		DON'T KNOW	Z
319	When did you take antibiotics for the last time?	[ ][ ] DAYS AGO	
		OR DATE OF LAST INTAKE:	
	FORMAT DD/MM/YYYY<br>*UNKN: 99/99/9999*	[ ][ ] / [ ][ ] /201 [ ]	
320	DID THE PATIENT PRESENT WITH VACCINATION BOOKLET?	YES	1
		NO	2
		NOT REPORTED	9
321	Have you been vaccinated against **Pneumococcus (PCV)**?	YES, 7-VALENT CONJUGATE	1
		YES, 13-VALENT CONJUGATE	2
		YES, 23-VALENT CONJUGATE	3
		YES, DON'T KNOW WHICH ONE	4
		NO	5 → GO TO 323
		DON'T KNOW	9 → GO TO 323
322	When have you been vaccinated against pneumococcus?	[ ][ ] [ ] MONTHS AGO [ ] YEARS AGO	
		OR DATE OF LAST VACCINE DOSE:	
	FORMAT DD/MM/YYYY<br>*UNKN: 99/99/9999*	[ ][ ] / [ ][ ] / [ ][ ][ ][ ]	
323	Have you been vaccinated against **Diphteria-Hepatitis B-Haemophilus (DTC-HepB-Hib)**?	YES	1
		NO	2 → GO TO 325
		DON'T KNOW	9 → GO TO 325
324	When have you been vaccinated against DTC-HepB-Hib?	[ ][ ] [ ] MONTHS AGO [ ] YEARS AGO	
		OR DATE OF LAST VACCINE DOSE:	
	FORMAT DD/MM/YYYY<br>*UNKN: 99/99/9999*	[ ][ ] / [ ][ ] / [ ][ ][ ][ ]	
325	Have you been vaccinated against **flu**?	YES	1
		NO	2 → GO TO 401
		DON'T KNOW	9 → GO TO 401

326	When have you been vaccinated against flu?<br><br>*FORMAT DD/MM/YYYY*<br>*DON'T KNOW: 99/99/9999*	[ ][ ] [ ] MONTHS AGO<br>[ ] YEARS AGO<br>OR DATE OF LAST VACCINE DOSE:<br>[ ][ ] / [ ][ ] / [ ][ ][ ][ ]

4. SOCIODEMOGRAPHIC DATA

401	Where are you currently living?<br>*NAME OF VILLAGE/CITY*	. . . .
402	What is your citizenship?<br><br>*CONSIDERED BY INTERVIEWEE AS MAIN OR PRIMARY CITIZENSHIP IF ≥2 CITIZENSHIPS*	LEBANESE .... 1<br>SYRIAN .... 2<br>PALESTINIAN .... 3<br>OTHER ________ 8<br>*(DETAIL)*<br>DON'T WANT TO SAY .... 9<br>DON'T KNOW 99
403	What kind of housing do you live in?	INDEPENDENT HOUSE/APPARTMENT NOT SHARED .... 11<br>INDEPENDENT HOUSE/APPARTMENT SHARED .... 12<br>SUBSTANDARD SHELTER (FACTORY, SHOP, GARAGE,…) 21<br>UNFINISHED OR ABANDONED BUILDING .... 22<br>COLLECTIVE SHELTER (≥6 FAMILIES) .... 31<br>PREFAB .... 32<br>TENT .... 33<br>NO SHELTER (HOMELESS) .... 41<br><br>OTHER ________ 81<br>*(DETAIL)*<br>DON'T WANT TO SAY .... 98<br>DON'T KNOW 99
404	How many rooms do your housing have?	[ ][ ] ROOMS, EXCLUDING TOILETS AND BATHROOMS<br>*(COLLECTIVE/SHARED HOUSING: ROOMS OCCUPIED BY HOUSEHOLD)*
405	What kind of toilets/latrines are there?	OWN TOILET .... 1<br>SHARED TOILET .... 2<br>OPEN AIR .... 3<br>DON'T WANT TO SAY .... 7<br>DON'T KNOW 9
406	What are your most important sources of water in your household?	PIPED .... 1<br>PRIVATE VENDOR .... 2<br>MUNICIPALITY, INFORMAL .... 3<br>UN AGENCY, NGO ASSISTANCE .... 4<br>SHOP, MARKET .... 5<br>PRIVATE WELL 6<br><br>OTHER ________ 8<br>*(DETAIL)*<br>DON'T KNOW 9
407	Do you burn oil or coal or wood or waste, which creates smoke in your house?	YES .... 1<br>NO .... 2<br>DON'T KNOW 9
408	How many people belong to your household?	[ ][ ] PEOPLE<br>*DON'T KNOW: 99*

And among these people:

409	How many children less than 5 years old are there?	[ ][ ]	CHILDREN 0-4 YEARS OLD<br>*NONE: 00 - DON'T KNOW: 99*
410	How many children aged more than 5 years are there?	[ ][ ]	CHILDREN 5-17 YEARS OLD, INCLUDING [ ][ ] ATTENDING SCHOOL<br>*LEAVE BLANK IF PREVIOUS ANSWER WAS 00*
411	How many adults less than 50 years old are there?	[ ][ ]	ADULTS 18-49 YEARS OLD<br>*NONE: 00 - DON'T KNOW: 99*
412	How many adults aged 50 or more are there?	[ ][ ]	ADULTS ≥50 YEARS OLD<br>*NONE: 00 - DON'T KNOW: 99*

5. CLINICAL EXAMINATION & COMPLEMENTARY EXAMS

501	OXYGEN SATURATION (SaO2)?<br>*NOT REPORTED: 999*	[ ][ ][ ] % ON ROOM AIR .... 1<br>WITH SUPPLEMENTAL O2 ..... 2<br>UNKNOWN 3
502	TEMPERATURE	[ ][ ] . [ ] °C AXILLARY .... 1<br>RECTAL .... 2<br>ORAL .... 3<br>TYMPANIC .... 4<br>OTHER ________________ 8<br>*(DETAIL)*
503	WEIGHT<br>*(IN CHILDREN)*	[ ][ ][ ] KG<br>*NOT REPORTED: 99*
504	HEIGHT<br>*(IN CHILDREN)*	[ ][ ][ ] CM<br>*NOT REPORTED: 999*
505	HEART RATE	[ ][ ][ ] CYCLES PER MINUTE<br>*NOT REPORTED: 999*

FOR CASES CONTINUE TO 506 - FOR CONTROLS, SKIP AND GO TO END

506	BLOOD PRESSURE	SYSTOLIC [ ][ ][ ] mmHg<br>*(NOT ASSESSED: 999)*<br>DIASTOLIC [ ][ ][ ] mmHg<br>*(NOT ASSESSED: 999)*
507	HAS A **CHEST X-RAY** BEEN PERFORMED?	YES .... 1<br>NO .... 2 → GO TO END<br>DON'T KNOW .... 9 → GO TO END
508	WHAT WAS THE RESULT?<br>*CHECK ALL THAT APPLY*	GENERALIZED DENSE HOMOGENOUS OPACIFICATION ..... A<br>INTERSTITIAL SYNDROME .... B<br>ALVEOLAR INFILTRATE .... C<br>ABSCESS .... D<br>PNEUMOTHORAX .... E<br>PLEURAL EFFUSION WITH PARENCHYMATOUS INFILTRATE ..... F<br>PLEURAL EFFUSION WITHOUT PARENCH. INFILTRATE .... G<br>OTHER ________________ X<br>*(DETAIL)*<br>NOT REPORTED .... Z
509	HAS IT BEEN READ BY A RADIOLOGIST?	YES .... 1<br>NO .... 2<br>DON'T KNOW 9
510	WHAT WAS THE PATIENT'S OUTCOME?	RETURNED HOME WITHOUT COMPLEMENTARY EXAMS 1<br>REFERRED FOR COMPLEMENTARY EXAMS .... 2<br>REFERRED FOR HOSPITALIZATION .... 3<br>OTHER ________________ 8<br>*(DETAIL)*

رقم تعريف المريض الذي شملته الدراسة | | | | | | |

1. معلومات عامة

101	تاريخ الزيارة<br>اليوم/ الشهر/ السنة - مثل: 99/99/9999	__ __ / __ __ / __ __ __ __
102	اسم محقق الدراسة<br>الشخص الذي يملأ هذه الاستمارة	....
103	اسم الطبيب المعالج	....
104	رقم ملف المريض لدى المركز<br>(إذا وجد)	....
105	مسجّل كحالة مرضية او كحالة غير مرضية للمقارنة	حالة مرضية .... 1<br>حالة غير مرضية للمقارنة .... 2<br>حالة مرضية مع أنبوب الدم تيمبوس (TEMPUS) .... 3<br>حالة غير مرضية للمقارنة مع أنبوب الدم تيمبوس (TEMPUS) 4

2. التقييم الطبي من قبل الطبيب

201	معدّل التنفس	__ __ دورة في الدقيقة<br>غير مذكور:99
202	معدّل التنفس (التقييم الثاني عند الأطفال الذين لم يتجاوزوا الخمس سنوات)	__ __ دورة في الدقيقة<br>غير مذكور:99
203	هل من نوع من مرض مزمن يؤثر على المريض؟<br>*حدّد بإشارة ما ينطبق عليك*<br>*يمكن تعبئة هذا القسم على أساس ملف المريض، إذا كان متوفرًا وكاملاً*	مرض القلب ________ A<br>(بالتفصيل)<br>مرض السكري .... B<br>ضغط الدم .... C<br>الفشل الكلوي .... D<br>مرض في الكبد .... E<br>الربو .... F<br>مرض السل .... G<br>فيروس نقص المناعة البشري / الإيدز .... H<br>مرض الروماتيزم .... I<br>مرض عصبي مزمن .... J<br>سكتة دماغية، وشلل نصفي، وشلل نصف سفلي .... K<br>سرطان، سرطان الدم، سرطان الغدد الليمفاوية .... L<br>مرض الانسداد الرئوي المزمن (COPD) .... M<br>آخر ________ X<br>(التفاصيل)<br>عدم وجود حالة مزمنة .... Y<br>لا أعلم .... Z
204	هل تتم معالجتك بواسطة ادوية كابتة للمناعة؟<br>يمكن تعبئة هذا القسم على أساس ملف المريض، إذا كان متوفرًا وكاملاً	لا .... 1<br>نعم، استنشاق الستيرويدات القشرية .... 2<br>نعم، الستيرويدات القشرية الفموية .... 3<br>نعم، العلاج الكيميائي .... 4<br>نعم، غيرها ________ 8<br>(التفاصيل)<br>لا أعلم 9

للحالات الرجاء الانتقال إلى 205 - و لحالة غير مرضية، الرجاء مراجعة 301

205 هل أظهر المريض العلامات التالية في فحص الصدر؟	A ..... LOWER CHEST WALL INDRAWING .... حركة الصدر السفلي<br>B .... ألم في الصدر<br>C ..... DULLNESS TO PERCUSSION .... أصمية تجاه الإيقاع<br>D ..... DIMISHED BREATH SOUNDS .... انخفاض أصوات التنفس<br>E ..... RASPING .... صرير<br>F ..... FINE, HIGH PITCHED CRACKLES خراخر ضارية مرتفعة ناعمة<br>G ..... COARSE, LOW PITCHED CRACKLES خراخر خشنة ضارية منخفضة<br>H ..... RONCHI .... حسيس غطيطي<br>I ..... LOW PITCHED WHEEZES .... وزيز منخفض الضارية<br>J EXPIRATORY, HIGH PITCHED WHEEZES وزيز زفيري مرتفع الضارية<br>X ______________________ آخر<br>(التفاصيل)<br>Y .... لا عوارض<br>Z .... لا أعلم

206 هل يُظهر المريض الأعراض التالية أو يشكو منها؟		نعم	كلا	(لم يتمّ تقييمه)
	11 عدوى الجهاز التنفسي العلوي (اعراض زكامية, سيلان الانف, التهاب البلعوم, التهاب الحلق, التهاب الاذن, التهاب الملتحمة)	1	2	9
	12 طفح الجلدي	1	2	9
	13 التهاب الغدد الليمفاوية	1	2	9
	14 ألم عضلي، ألم مفصلي	1	2	9
	21 نعاس زائد او اغماء	1	2	9
	22 اختلاج	1	2	9
	23 عدم القدرة على الشرب	1	2	9
	24 التقيؤ المستمر	1	2	9
	25 صرير تنفسي اثناء هدوء الطفل	1	2	9
	26 سوء التغذية الحاد	1	2	9

207 هل وصف المعاين للمريض مضادات حيوية؟	نعم .... 1<br>كلا .... 2 ← مراجعة 301<br>لا أعلم .... 9 ← مراجعة 301

208 ما هي المضادات الحيوية التي سوف يتناولها المريض؟<br><br>أشر إلى كل ما ينطبق عليك	A AMOXICILLIN .... أموكسيسلين<br>B AMOXI+CLAV (AUGMENTIN) (أوغمونتان) أموكسيسلين + حمض كلافولانيك<br>C CEFIXIME .... سيفيكسيم<br>D CEFUROXIME .... سيفوروكسيم<br>E CEFTRIAXONE .... سيفترياكسون<br>F LEVOFLOXACIN .... ليفوفلوكساسين<br>G CIPROFLOXACIN .... سيبروفلوكساسين<br>H AZITHROMYCIN .... أزيثروميسين<br>I CLARITHROMYCIN .... كلاريثروميسين<br>J COTRIMOXAZOLE .... كوتريموكسارول<br>K METRONIDAZOLE +/- SPIRAMYCIN ميترونيدازول +/- سبيراميسين<br>X ______________________ آخر<br>(التفاصيل)<br>Z .... اسم غير معروف<br>الجرعة [ ][ ][ ][ ] ملغ/اليوم<br>المدة [ ][ ] يوم

3. التاريخ الطبي

301 عمر المريض	السنوات [ ][ ] إذا أقل من سنة واحدة من العمر: [ ][ ] الأشهر
302 تاريخ الولادة<br>اليوم/ الشهر/ السنة - مثل: 99/99/9999	[ ][ ] / [ ][ ] / [ ][ ][ ][ ]
303 جنس المريض	ذكر .... 1 ← مراجعة 305<br>أنثى .... 2 ←

للمرضى البالغين، الرجاء الانتقال إلى 304 - للأطفال، الرجاء مراجعة 307

304 هل أنت حامل؟<br>للنساء في سن الإنجاب فقط	نعم .... 1<br>كلا .... 2<br>غير قابل للتطبيق .... 3<br>لا أعرف / لا أريد أن أجيب 9
305 هل أنت مدخن؟	نعم .... 1<br>كلا .... 2 ← مراجعة 311<br>لا أعرف / لا أريد أن أجيب .... 9 ← مراجعة 311
306 ما هو عدد السجائر/الشيشة التي تدخّنها؟<br>لا أدخّن: 00<br>لا أعلم: 99	سيجارة [ ][ ] يوميًا [ ] أسبوعيًا [ ]<br>شيشة [ ] يوميًا [ ] أسبوعيًا [ ]

للأطفال الرضع أقل من 1 سنة الرجاء الانتقال إلى 307 - ، للأطفال الأكبر سنًا والبالغين، الرجاءمراجعة 311

307 هل ولد الطفل قبل أوانه؟<br>طفل مولود قبل أوانه = فترة الحمل أقل من 37 أسبوعًا	نعم .... 1<br>كلا .... 2<br>لا أعلم 9
308 ما هو وزن الطفل عند الولادة؟<br>مجهول: 9.9	[ ] . [ ] كيلوغرام
309 هل يتم فقط إرضاع الطفل؟	نعم .... 1<br>كلا .... 2<br>لا أعلم 9
310 هل يعتبر نمو الطفل مناسبًا لعمره؟<br>(التقييم العياديّ)	نعم .... 1<br>كلا .... 2<br>لا أعلم 9

لحالة غير مرضية الرجاء الانتقال إلى 311 - وللحالات، الرجاء مراجعة 315

311 ما هو السبب الرئيسي (العارض) الذي جعلك تأتي إلى المركز الصحي؟<br>الاختيار فقط خيار واحد	امراض الجهاز التنفسي .... 11<br>امراض الجهاز الهضمي .... 12<br>مرض حموي .... 13<br>مرض جلدي .... 14<br>مرض بالظهر او بالمفاصل .... 15<br>حادث, اصابة خارجية .... 16<br>التلقيح .... 21<br>الحمل(زيارة ما قبل الولادة) .... 22<br>مراجعة الأمراض المزمنة (مرض السكري، وضغط الدم، اضطراب في ضربات القلب، الفشل الكلوي ,...) .... 23<br>آخر ________ (الاسم) 80<br>مرافقة أحد أفراد الأسرة .... 91<br>لا أعرف / لا أريد أن أجيب 99
312 هل تمّ إدخالك المستشفى في الأشهر الثلاثة الماضية ؟<br>لا يقل عن 2 يوماء في غضون 90 يومًا	نعم .... 1<br>كلا .... 2<br>لا أعلم 9
313 هل تم رعاية طبية اي جروح خلال الشهر الماضي؟<br>في غضون 30 يومًا	نعم .... 1<br>كلا .... 2<br>لا أعلم 9
314 هل تعيش في دار للمسنين أو مركز للرعاية على المدى الطويل؟	نعم .... 1<br>كلا .... 2<br>لا أعلم 9

315	هل يدخّن أحدًا في منزلك؟	نعم .... 1<br>كلا .... 2<br>لا أعلم 9
316	هل تشعر حاليًا بالحمى أو هل شعرت بالحمى في الأسبوعين الماضيين؟	نعم .... 1<br>كلا .... 2<br>لا أعلم 9
317	هل تم معالجتك بالمضادات الحيوية خلال الشهر الماضي؟	نعم .... 1<br>كلا .... 2 ← مراجعة 320<br>غير قابل للتطبيق .... 3 ← مراجعة 320<br>لا أعلم .... 9 ← مراجعة 320
318	ما هو المضاد الحيوي الذي تناولته؟<br><br>حدّد بإشارة ما ينطبق عليك	أموكسيسلين .... AMOXICILLIN A<br>أموكسيسلين + حمض كلافولانيك (أوغمونتان) AMOXI+CLAV (AUGMENTIN) B<br>سيفيكسيم .... CEFIXIME C<br>سيفوروكسيم .... CEFUROXIME D<br>سيفترياكسون .... CEFTRIAXONE E<br>ليفوفلوكساسين .... LEVOFLOXACIN F<br>سيبروفلوكساسين .... CIPROFLOXACIN G<br>أزيثروميسين .... AZITHROMYCIN H<br>كلاريثروميسين .... CLARITHROMYCIN I<br>كوتريموكسارول .... COTRIMOXAZOLE J<br>ميترونيدازول +/- سبيراميسين METRONIDAZOLE +/- SPIRAMYCIN K<br>آخر ________ X<br>(التفاصيل)<br>لا أعلم .... Z
319	متى تناولت المضادات الحيوية للمرة الأخيرة؟<br><br>اليوم/ الشهر/ السنة<br>مثل: 99/99/9999	منذ [ ][ ] يوم<br>أو تاريخ آخر مرّة:<br>[ ][ ] / [ ][ ] / [ ][ ][ ][ ]
320	هل أحضر المريض كتيب التلقيح؟	نعم .... 1<br>كلا .... 2<br>غير مذكور 9
321	هل تم تلقيحك ضد *Streptococcus pneumoniae* (PCV)؟	نعم, (PCV7) .... 1<br>نعم, (PCV13) .... 2<br>نعم, (PCV23) .... 3<br>نعم، لا أعلم اسم اللقاح .... 4<br>كلا .... 5 ← مراجعة 323<br>لا أعلم .... 9 ← مراجعة 323
322	متى تم تلقيحك ضد المكورات الرئوية؟<br><br>اليوم/ الشهر/ السنة<br>مثل: 99/99/9999	منذ [ ][ ] [ ] أشهر / [ ] سنة<br>أو تاريخ آخر جرعة تلقيح:<br>[ ][ ] / [ ][ ] / [ ][ ][ ][ ]
323	هل تمّ تلقيحك ضدّ الخُناق والتهاب الكبد الوبائي والهيموفيلوس نوع ب (DTC-HepB-Hib)؟	نعم .... 1<br>كلا .... 2 ← مراجعة 325<br>لا أعلم .... 9 ← مراجعة 325
324	متى تمّ تلقيحك ضدّ DTC-HepB-Hib؟<br><br>اليوم/ الشهر/ السنة<br>مثل: 99/99/9999	منذ [ ][ ] [ ] أشهر / [ ] سنة<br>أو تاريخ آخر جرعة تلقيح:<br>[ ][ ] / [ ][ ] / [ ][ ][ ][ ]
325	هل تمّ تلقيحك ضد الأنفلونزا؟	نعم .... 1<br>كلا .... 2 ← مراجعة 401<br>لا أعلم .... 9 ← مراجعة 401

326	متى تمّ تلقيحك ضد الأنفلونزا؟<br>اليوم/ الشهر/ السنة<br>مثل: 99/99/9999	منذ [ ][ ] أشهر / سنة<br>أو تاريخ آخر جرعة تلقيح: [ ][ ] / [ ][ ] / [ ][ ][ ][ ]

4. البيانات الاجتماعية والديموغرافية

401	أين تعيش حاليًا؟<br>اسم القرية / المدينة	....
402	ما هي جنسيتك؟<br>تعتمد الجنسية التي يعتبرها المريض جنسيّته الرئيسيّة	لبناني ..... 1<br>سوري ..... 2<br>فلسطيني ..... 3<br>جنسية أخرى ______ 8<br>(تفاصيل)<br>لا أريد الإجابة ..... 9<br>لا أعلم 99
403	ما هو نوع السكن الذي تعيش فيه؟	منزل مستقل / شقة غير مشتركة ..... 11<br>منزل مستقل / شقة مشتركة ..... 12<br>مأوى دون المستوى المطلوب (مصنع، متجر، مرآب) ..... 21<br>مبنى غير مكتمل أو مهجور ..... 22<br>مأوى جماعي (ست عائلات أو أكثر) ..... 31<br>منزل جاهز ..... 32<br>خيمة ..... 33<br>من دون مأوى ..... 41<br>آخر ______ 81<br>(تفاصيل)<br>لا أريد الإجابة ..... 98<br>لا أعلم 99
404	ما هو عدد غرف مسكنك؟	[ ][ ] غرفة، باستثناء المراحيض والحمامات<br>(مسكن جماعي /مشترك: يقيم أهل البيت في الغرف)
405	ما هو نوع المراحيض؟	مرحاض خاص ..... 1<br>مرحاض مشترك ..... 2<br>في الهواء الطلق ..... 3<br>لا أريد الإجابة ..... 7<br>لا أعلم 9
406	ما هي أهم مصادر المياه في المنزل؟	مياه الشفة الرسمية ..... 1<br>بائع خاص ..... 2<br>بلدية غير رسمية ..... 3<br>وكالة تابعة للأمم المتحدة، أومساعدة من المنظمات غير الحكومية ..... 4<br>متجر أو سوق ..... 5<br>بئر خاص ..... 6<br>آخر ______ 8<br>(تفاصيل)<br>لا أعلم 9
407	هل تشعل زيوت, فحم, حطب, او النفايات مما ينتج الدخان في منزلك؟	نعم ..... 1<br>كلا ..... 2<br>لا أعلم 9
408	ما هو عدد الأشخاص االذين ينتمون إلى منزلك؟	[ ][ ] شخصًا<br>لا أعلم: 99

لنصف هؤلاء الأشخاص أكثر:

409 ما هو عدد الأطفال الذين هم ما دون 5 سنوات؟	☐☐ طفلا 0-4 سنوات من العمر<br>لا أحد: 00 لا أعلم: 99
410 ما هو عدد الأطفال الذين هم أكبر من 5 سنوات؟	☐☐ طفلا 5 – 17 سنة، منهم ☐☐ يذهبون إلى المدرسة<br>اتركه فارغًا إذا كانت الإجابة السابقة 00
411 ما هو عدد البالغين الذين هم ما دون 50 عامًا؟	☐☐ بالغًا 18 – 49 سنة<br>لا أحد: 00 لا أعلم: 99
412 ما هو عدد البالغين الذين هم أكثر من 50 عامًا؟	☐☐ بالغًا ما فوق ال 50 سنة<br>لا أحد: 00 لا أعلم: 99

5. فحص سريري وفحوصات تكميلية

501 تشبع الاكسجين	☐☐☐ %<br>غير مذكور: 999<br>هواء الغرفة .... 1<br>مع الأكسجين الإضافي .... 2<br>غير معروف 3
502 درجة حرارة الجسم	☐ . ☐☐ درجة مئوية<br>إبطي .... 1<br>شرجي .... 2<br>فموي .... 3<br>أذني .... 4<br>آخر ____________ 8<br>(تفاصيل)
503 الوزن<br>للأطفال	☐☐☐ كلغ<br>غير مذكور:99
504 الطول<br>للأطفال	☐☐☐ سم<br>غير مذكور:999
505 معدل ضربات القلب	☐☐☐ ضربة في الدقيقة<br>غير مذكور:999

للحالات الرجاء الانتقال إلى 506 - و لحالة غير مرضية، الرجاء توجّه النهاية

506 ضغط الدم	انقباضي ☐☐☐ مم الزئبق<br>(لم يتمّ تقييمه: 999)<br>انبساطي ☐☐☐ مم الزئبق<br>(لم يتمّ تقييمه: 999)
507 هل قمت بتصوير الصدر بالاشعة السينية ؟	نعم .... 1<br>كلا .... 2 ← مراجعة 510<br>لا أعلم .... 9 ← مراجعة 510
508 ما هي النتيجة؟<br>أشر إلى كل ما ينطبق عليك	عتامة القرنية المتجانسة الكثيفة والعامة<br>A ..... GENERALIZED DENSE HOMOGENOUS OPACIFICATION<br>B ..... INTERSTITIAL SYNDROME .... متلازمة الخلالي<br>C ..... ALVEOLAR INFILTRATE .... تسلّل السنخية<br>D ..... ABSCESS .... خرّاج<br>E ..... PNEUMOTHORAX .... الاسترواح الصدري<br>الانصباب الجنبي مع التسلل المتني<br>F ..... PLEURAL EFFUSION WITH PARENCHYMATOUS INFILTRATE<br>الانصباب الجنبي من دون التسلل المتني<br>G ..... PLEURAL EFFUSION WITHOUT PARENCHYMATOUS INFILTRATE<br>X ____________ آخر<br>(تفاصيل)<br>Z .... غير مذكور
509 هل قرأ طبيب الأشعة النتيجة؟	نعم .... 1<br>كلا .... 2<br>لا أعلم 9
510 ما هي نتيجة المريض؟	العودة إلى المنزل بلا المزيد من الفحوصات .... 1<br>الإحالة إلى المزيد من الفحوصات .... 2<br>الإحالة إلى دخول المستشفى للعلاج .... 3<br>آخر ____________ 8<br>(تفاصيل)
