# Supplementary material for: Investigating Pneumonia Etiology Among Refugees and the Lebanese population (PEARL): A study protocol
Source: Gates Open Res. 2019 Jun 13;2:19. Originally published 2018 Apr 18. [Version 2] doi: 10.12688/gatesopenres.12811.2 (PMC7569241; doi:10.12688/gatesopenres.12811.2)
Supplement: Peer review history

# Peer review: Reviewer response for version 2

**DOI:** 10.21956/gatesopenres.14036.r29789

**Published:** 2020-10-13

**Authors:** Carina King

**Affiliations:** 1 Department of Global Public Health, Karolinska Institutet, Stockholm, Sweden

This presents a clear study protocol, aiming to answer an important (but challenging!) question - of LRTI aetiology in a humanitarian setting. 

 Background:

- Can you also provide information on vaccine coverage (if it can be disaggregated?), and influenza vaccine policies? 
- Indicate what formulation of PCV.

Protocol:

- Why was chest indrawing only considered for children <=3 years?
- Can you provide a bit more information on whether pulse oximetry was new in this setting, and if so, what training was done and quality control?
- It wasn't clear whether you intend to use the averaged RR over two counts, or just one. And whether this will be done for all age groups. 
- Will any analyses of multi-pathogen versus single pathogen presentation be done? Thinking of the PERCH findings, where this was an important result? 

Is the study design appropriate for the research question?

Yes

Is the rationale for, and objectives of, the study clearly described?

Yes

Are sufficient details of the methods provided to allow replication by others?

Yes

Are the datasets clearly presented in a useable and accessible format?

Not applicable

Reviewer Expertise:

Infectious epidemiology, paediatric pneumonia

I confirm that I have read this submission and believe that I have an appropriate level of expertise to confirm that it is of an acceptable scientific standard.

# Peer review: Reviewer response for version 2

**DOI:** 10.21956/gatesopenres.14036.r27382

**Published:** 2020-01-14

**Authors:** Anne-Laure Page

**Affiliations:** 1 Epicentre, Paris, France

I have reviewed the response from the authors and believe that the revisions are appropriate.

Is the study design appropriate for the research question?

Yes

Is the rationale for, and objectives of, the study clearly described?

Yes

Are sufficient details of the methods provided to allow replication by others?

Partly

Are the datasets clearly presented in a useable and accessible format?

Not applicable

Reviewer Expertise:

NA

I confirm that I have read this submission and believe that I have an appropriate level of expertise to confirm that it is of an acceptable scientific standard.

# Peer review: Reviewer response for version 1

**DOI:** 10.21956/gatesopenres.13878.r26432

**Published:** 2018-11-26

**Authors:** Stephen R. Howie

**Affiliations:** 1 Department of Paediatrics: Child and Youth Health, University of Auckland, Auckland, New Zealand

Thank you for the opportunity to review this study protocol. This protocol describes a multicentre case control study of pneumonia etiology in refugee populations in Lebanon. This is a population at high risk of pneumonia and data on etiology in this group will be welcome. The study has already completed recruitment, and is undergoing analysis. It is no simple task undertaking such a study, and I look forward to seeing its findings.

 I have a number of comments and suggestions. The protocol is clear in some of its aspects and unclear in others: I commend the authors for the former and I will focus my comments on the latter, which are points to be addressed.

 The authors rightly stress the limitations of ‘blindly’ applying etiology data from other studies to this population. They should be similarly cautious about applying their findings to those suffering in the midst of humanitarian crisis elsewhere, and even to those in the study settings at other times.  Fifteen months is relatively short, and refugee contexts are unstable and prone to epidemics. What happens in that 15 month period may not be what is happening the next year.  A case-control study such as this certainly has value, and longitudinal surveillance would add more value. The relationship between existing pneumococcal surveillance systems in Lebanon and this study is not clear from the protocol; adding broader pathogen surveillance to those systems has much to recommend it. I note too the lack of any data about TB in the study protocol, a gap it would be good to fill. Are there plans to address this in the future?

 The protocol makes much of the benefits of transcriptomic analysis (including that it ‘reduces the need to identify pathogens by culturing respiratory tract samples’) but is relatively silent on how this will be undertaken and how data will be used to achieve the primary aims.  Biomarkers for pneumonia etiology (whether they be transcriptomic or other), while attractive conceptually, have many limitations, and their practical applicability at this point need careful scrutiny. I wonder whether the transcriptomic aspect of this study is an exploratory add-on (not in itself an invalid thing to do). I see no mention of power calculations with respect to the transcriptomic element, for instance, or how it will be integrated into the analysis as a whole.  

 The power calculation description is restricted to the primary objective. It would be helpful to show that there is adequate power for all objectives. 

 The analytic methods planned for the primary objective are fairly restricted.  I would encourage the authors to learn from the approaches taken in studies such as PERCH and get the most they can out of the data. This can still be done even though recruitment has been completed.

 There is no mention of using a point-of-care testing approach except at the end of the objectives and I wonder what was meant by that.

 The case definition used was not explicitly linked to the WHO definition, but reference was made to using WHO categorisation of severity.   Clarifying the relationship between study and WHO definitions would be helpful.  

 What does the protocol mean when it says that inclusion requires “no wheezing suggestive of asthma at auscultation”? What sort of wheezing is that? Does ‘suggestive’ mean age, history, trial of bronchodilator, or something else?  What does the protocol mean when it specifies “particular attention to pneumonia with a breathing rate > 50 breaths per minute’? Was this a de facto entry criterion? 

 What does the protocol mean when it says that ‘relevant’ results will be communicated to clinicians. Which are these? Did you share NP or sputum PCR results and if so how did you suggest that clinicians interpret them?

 The protocol appears to state that 6ml of blood will be taken for blood culture. Even in infants? The sample collection protocols requires clarification here.

Is the study design appropriate for the research question?

Partly

Is the rationale for, and objectives of, the study clearly described?

Yes

Are sufficient details of the methods provided to allow replication by others?

Partly

Are the datasets clearly presented in a useable and accessible format?

Not applicable

Reviewer Expertise:

NA

I confirm that I have read this submission and believe that I have an appropriate level of expertise to confirm that it is of an acceptable scientific standard, however I have significant reservations, as outlined above.

# Response

**Published:** 2019-03-01

**Authors:** Thomas Kesteman

**Affiliations:** Fondation Mérieux, France

We would like to express thanks the reviewer for his useful comments and suggestions. We carefully reviewed his report and we changed the manuscript according to his propositions. Our answers to his questions and comments are listed in the text below.

**Comment:** The authors rightly stress the limitations of ‘blindly’ applying etiology data from other studies to this population. They should be similarly cautious about applying their findings to those suffering in the midst of humanitarian crisis elsewhere, and even to those in the study settings at other times. 

**Action/Answer**: We added a sentence in the paragraph regarding the limitations of the study ( *Discussion* chapter): “although the situation of Syrian refugees in Lebanon shares a lot of characteristics with other crises, one should be cautious about applying the results of PEARL to other humanitarian crises, because of the variations of the epidemiology of LRTI.”

**Comment:** Fifteen months is relatively short, and refugee contexts are unstable and prone to epidemics. What happens in that 15 month period may not be what is happening the next year.  A case-control study such as this certainly has value, and longitudinal surveillance would add more value. The relationship between existing pneumococcal surveillance systems in Lebanon and this study is not clear from the protocol; adding broader pathogen surveillance to those systems has much to recommend it.

**Action/Answer**: We added a sentence in the *Discussion, *just before the one above: “Finally, the external validity of our study is limited in time (15 months) and space (four health facilities). A case-control study cannot replace longitudinal surveillance systems, as are currently being set up in Lebanon, e.g. the Lebanese Inter-Hospital Pneumococcal Surveillance Program.”.

**Comment:** I note too the lack of any data about TB in the study protocol, a gap it would be good to fill. Are there plans to address this in the future?

**Action/Answer**: This was acknowledged as a limitation of our study in the first version of the manuscript: “certain aspects of CAP will not be explored in this study […]. Patients with tuberculosis are expected to be excluded, since duration of symptoms >14 days is an exclusion criterion.” Nevertheless, we added the following sentence: “The incidence rate of tuberculosis among Syrian refugees did not increase in the last couple of years and remains relatively low at close to pre-war levels, i.e. 10-20/100.000 population. Therefore, we consider it unlikely that tuberculosis is a major cause of acute LRTI in our population.” and we provided references for this assertion.

**Comment:** The protocol makes much of the benefits of transcriptomic analysis (including that it ‘reduces the need to identify pathogens by culturing respiratory tract samples’) but is relatively silent on how this will be undertaken and how data will be used to achieve the primary aims.  Biomarkers for pneumonia etiology (whether they be transcriptomic or other), while attractive conceptually, have many limitations, and their practical applicability at this point need careful scrutiny. I wonder whether the transcriptomic aspect of this study is an exploratory add-on (not in itself an invalid thing to do). I see no mention of power calculations with respect to the transcriptomic element, for instance, or how it will be integrated into the analysis as a whole.

**Action/Answer**: The reviewer is right: transcriptomics is an add-on, as we have clearly stated that “Patient enrollment began in November 2016” and that “Sampling for transcriptomics was initiated in May 2017” (i.e. 7 months after the initiation of the study). Therefore, the overall study has not been powered to meet this secondary objective.

 In order to clarify these points, we added the following sentence in the chapter related to Data management and analysis: “Etiologic fractions will be also computed from transcriptomics data, independently from calculations from microbiological data, and compared with each other.”

**Comment:** The power calculation description is restricted to the primary objective. It would be helpful to show that there is adequate power for all objectives.

**Action/Answer**: The study has been powered for the primary objective only, and the uncertainty around the values corresponding to secondary objectives will be provided by 95% confidence intervals. We added the following sentence in *Data management and analysis*: “Ninety-five-percent confidence intervals will be calculated, with regards to the outcomes linked to the primary and secondary objectives.”

**Comment:** The analytic methods planned for the primary objective are fairly restricted.  I would encourage the authors to learn from the approaches taken in studies such as PERCH and get the most they can out of the data. This can still be done even though recruitment has been completed.

**Action/Answer**: Methodologies such as partially latent class models (if this is what Dr Howie refers to) have been considered for PEARL study. Nevertheless, they can poorly be applied to our dataset given the restricted range of samples in our study: nasopharyngeal swab mainly, some sputum, and blood cultures -that are expected to have a low yield in primary health care and will therefore be poorly informative. In comparison with PERCH, where information from a much wider range of biological samples taken from severe cases was used, our dataset would be poorly suitable for complex models. Another drawback of PLC models is that they appear as a ‘blackbox’ while Population Attributable Fraction, resulting from Generalized Linear Models, are much more straightforward and comprehensible.  

**Comment:** There is no mention of using a point-of-care testing approach except at the end of the objectives and I wonder what was meant by that.

**Action/Answer**: Actually, the PoC tests will be performed in laboratories located nearby but not in the institution where the patients will be included.  The results are therefore not become available real time and cannot be considered true PoC tests.

**Comment:** The case definition used was not explicitly linked to the WHO definition, but reference was made to using WHO categorisation of severity.   Clarifying the relationship between study and WHO definitions would be helpful. 

**Action/Answer**: Exact. We added the following sentence in *Inclusion criteria*: “The case definition of CAP was based on the WHO’s IMCI. “

**Comment:** What does the protocol mean when it says that inclusion requires “no wheezing suggestive of asthma at auscultation”? What sort of wheezing is that? Does ‘suggestive’ mean age, history, trial of bronchodilator, or something else? 

**Action/Answer**: The reviewer is correct. This is indeed not clear. We added the following sentence in *Inclusion criteria*: “Characteristics of wheezing suggestive of asthma included: expiratory wheezing, high pitched wheezing, wheezing with history of asthma and without fever nor history of fever, and any clinical picture such as the clinician would exclude LRTI and retain asthma as final diagnostic.”

**Comment:** What does the protocol mean when it specifies “particular attention to pneumonia with a breathing rate > 50 breaths per minute’? Was this a de facto entry criterion?

**Action/Answer**: No, it just means that we measured the breathing rate twice in children, in order to evaluate its pertinence in our own dataset. In the manuscript, we therefore changed the sentence as follows: “Moreover, in children under five years of age, we will assess the breathing rate twice, since it has been shown that the cut-off of > 50 breaths per minute, assessed twice, is more specific for pneumonia than the standard IMCI recommendations for children 1–5 years old (40 breaths/min), and was successfully adopted in the ALMANACH protocol.”

**Comment:** What does the protocol mean when it says that ‘relevant’ results will be communicated to clinicians. Which are these? Did you share NP or sputum PCR results and if so how did you suggest that clinicians interpret them?

**Action/Answer**: The criteria for feedback of individual lab data, as specified in the protocol shared with laboratories, included:

- Identification of clinically relevant bacteria in blood (e.g. no bacteria associated to skin contamination, like coagulase-negative *Staphylococci*), by conventional microbiology or molecular tools. Information of identification and AST results will both be reported to the clinician by phone as soon as they will become available.
- Identification of clinically relevant bacteria in nasopharyngeal swab or sputum of CAP cases, by conventional microbiology or molecular tools. This includes detection of atypical bacteria ( *Chlamydia pneumoniae* or *Mycoplasma pneumoniae*) by BioFire. Information related to the identification and AST results will be reported by phone to the clinician as soon as they become available.
- Identification of RSV in infants (cases or controls) aged ≤12 months.
- Positive Binax test in urine of case.

This was added to the manuscript, in the chapter *Analysis of biological samples*.

**Comment:** The protocol appears to state that 6ml of blood will be taken for blood culture. Even in infants? The sample collection protocols requires clarification here.

**Action/Answer**: No, in infants and small children, the volume of blood taken was lower. The following sentence has been added to clarify this: “The target blood volume for blood culture is 1-3 mL in under-fives and 6 mL in children aged five or above and in adults, following manufacturers’ recommendations.”

# Peer review: Reviewer response for version 1

**DOI:** 10.21956/gatesopenres.13878.r26639

**Published:** 2018-09-28

**Authors:** Anne-Laure Page

**Affiliations:** 1 Epicentre, Paris, France

This manuscript describes the study protocol for a study on the etiology of pneumonia among refugees and local population in Lebanon. The study, which has already completed all inclusions, was designed to investigate the population-attributable fractions (PAF) of specific viral and bacterial pathogens by comparing the proportion of pathogens detected by culture, PCR and rapid tests among pneumonia cases and age-matched controls. In addition, it aims at describing the pathogens isolates (antimicrobial susceptibility and serotypes if applicable), identifying risk factors for pneumonia and providing transcriptomics data to identify expression patterns associated with specific pathogens. The manuscript is clear and well written and describes the study adequately, except for some areas, which would require additional information:  

1. Describe the recommended volumes of blood collection for blood culture according to the age
2. The study sites are primary healthcare facility (no hospital facility), but some of the inclusion criteria (in particular chest indrawing in children < 3 years of age) would require immediate referral to hospital. Please describe how inclusions are performed for these patients.
3. Describe the rationale for having gastrointestinal infections as exclusion criteria for controls.
4. The sample size calculation is stratified by age group (150 patients for each of four age groups), leading to a final sample size of minimum 600 cases and maximum 900 cases. It would be helpful to know what were the expected proportions of patients in each age group to understand the final sample size calculation. The authors could comment on the fact that finally 1420 patients were included, which is not consistent with the initial sample size calculated.
5. More information could be provided on the culture of bacterial from respiratory samples (sputum samples and nasopharyngeal swabs). Is quality of the sputum and absence of saliva contamination assessed? Which selective media are used?
6. Please provide more information about transcriptomics analysis (at least a reference).
7. The references cited for the semi-quantitative multiplex real-time PCR assay to detect *S. pneumoniae*, *S. aureus* and *H. influenzae* B do not provide sufficient information on the method. Authors should also describe more in details how this assay is performed on EDTA whole blood (volume of blood used for extraction and in the PCR assay). Interpretation of the PCR results from blood, sputum and nasopharyngeal swabs should also be described, in particular if there is a threshold (in Ct or copies/mL) used to consider the result as positive.
8. It is unclear whether the additional indicators described in the study outcomes would come from the study results or from other sources. Some, like clinical signs of CAP, can be analysed from the study data, whereas others, while for others, like agent-specific hospitalization and fatality rates, the corresponding data is not collected in the CRF.
9. The primary objective states that the PAFs will be estimated “using a combination of conventional methods and transcriptomics”, but it is unclear in the analysis plan if or how the transcriptomics data will be used to determine the PAFs and how these transcriptomics data will be analysed.

Is the study design appropriate for the research question?

Yes

Is the rationale for, and objectives of, the study clearly described?

Yes

Are sufficient details of the methods provided to allow replication by others?

Partly

Are the datasets clearly presented in a useable and accessible format?

Not applicable

Reviewer Expertise:

NA

I confirm that I have read this submission and believe that I have an appropriate level of expertise to confirm that it is of an acceptable scientific standard, however I have significant reservations, as outlined above.

# Response

**Published:** 2019-03-01

**Authors:** Thomas Kesteman

**Affiliations:** Fondation Mérieux, France

We would like to express thanks the reviewer for her useful comments and suggestions. We carefully reviewed her report and we changed the manuscript according to her propositions. These changes included adding the complete methodology of a triplex PCR that had not been disclosed in a previous scientific article. Therefore, we added M Stéphane Pouzol, who designed and developed that technique, as an author of the present manuscript. Our answers to her questions and comments are listed in the text below.

**Comment:** Describe the recommended volumes of blood collection for blood culture according to the age.

**Action/Answer:** The following sentence has been added to clarify this: “The total volume of blood taken is 0.5-1.0 mL in controls of all ages and, in cases, 2.5-5.0 mL in infants (<1 year old), 3.5-6.0 mL in children aged one to four, 10.5-17.0 and 6 mL in children aged five or above, and 18.5-19.0 mL in adults (≥18 years old), with a flexibility in the age cut-off depending on the individual’s weight and overall health status.”

**Comment:** The study sites are primary healthcare facility (no hospital facility), but some of the inclusion criteria (in particular chest indrawing in children < 3 years of age) would require immediate referral to hospital. Please describe how inclusions are performed for these patients.

**Action/Answer:** We added the following in *Study setting and design*: “After enrolment in the study, patients will be managed as per local guidelines and doctors’ recommendations; in particular, the study won’t alter procedures for referral for complementary examinations or hospitalization.”

**Comment:** Describe the rationale for having gastrointestinal infections as exclusion criteria for controls.

**Action/Answer:** We added the following in *Inclusion criteria - Controls* and provided a reference: “Individuals with gastro-intestinal symptoms were excluded as some viruses (adenovirus, enterovirus, and coronavirus) are proven or suspected to cause either respiratory or gastro-intestinal symptoms, and including such patients as controls would result in an underestimation of the attributable fraction of these pathogens.”

**Comment:** The sample size calculation is stratified by age group (150 patients for each of four age groups), leading to a final sample size of minimum 600 cases and maximum 900 cases. It would be helpful to know what were the expected proportions of patients in each age group to understand the final sample size calculation.

**Action/Answer:** At the moment of protocol design, there were no data available regarding incidence of lower respiratory tract infection in the different age groups. We assume that will would include an approximately equal number of cases in each of the four age groups. Hence, 150*4 = 600.

**Comment:** The authors could comment on the fact that finally 1420 patients were included, which is not consistent with the initial sample size calculated.

**Action/Answer:** Actually, this is a result of the study, and that should not have appeared in the protocol. Therefore, we removed that sentence. It will be discussed in the manuscript presenting the results of the study.

**Comment:** More information could be provided on the culture of bacterial from respiratory samples (sputum samples and nasopharyngeal swabs). Is quality of the sputum and absence of saliva contamination assessed? Which selective media are used?

**Action/Answer:** We agree with the reviewer’s comment. We modified the manuscript as follows: “Sputum samples and STGG media will be inoculated onto different selective agar plates according to standard laboratory procedures (at least one blood culture medium, one chocolate agar, and one medium selective for gram-negative bacilli) and incubated under specific conditions (37°C, aerobic atmosphere supplemented with CO _2_) to determine the presence of respiratory pathogens and assess antibiotic susceptibility. Quality of sputum and absence of contamination by saliva will be assessed by standard laboratory procedures.”

**Comment:** Please provide more information about transcriptomics analysis (at least a reference).

**Action/Answer:** Done. The *Transcriptomics* paragraph of *Analysis of biological samples* chapter and the *Data management and analysis* chapter have been substantially supplemented to include all procedures related to transcriptomics, and we added references to articles where those methods have been used.

**Comment:** The references cited for the semi-quantitative multiplex real-time PCR assay to detect S. pneumoniae, S. aureus and H. influenzae B do not provide sufficient information on the method.

**Action/Answer:** We amended references and added the information required for performing the PCR in the *Molecular biology testing of respiratory samples *paragraph of *Analysis of biological samples* chapter: sequences of primers, probes, reagents, and temperature cycle.

**Comment:** Authors should also describe more in details how this assay is performed on EDTA whole blood (volume of blood used for extraction and in the PCR assay).

**Action/Answer:** Done. The *Detection of bacteria in blood *paragraph of *Analysis of biological samples* chapter has been modified as follows: “Whole blood (200 μL EDTA) samples from all cases will be extracted using QIAamp DNA blood mini kit (Qiagen, Hilden, Germany), and the same semi-quantitative multiplex Real-Time PCR assay as for respiratory samples will be conducted on 5 μL of extracted DNA to identify *Streptococcus pneumoniae, Staphylococcus aureus* and *Haemophilus influenzae* B.”

**Comment:** Interpretation of the PCR results from blood, sputum and nasopharyngeal swabs should also be described, in particular if there is a threshold (in Ct or copies/mL) used to consider the result as positive.

**Action/Answer:** Done. The following sentence has been added in *Molecular biology testing of respiratory samples *paragraph of *Analysis of biological samples* chapter: “Amplification curves will be examined individually by two independent technicians, at the local laboratory and at the Laboratoire des Pathogènes Emergents (Lyon, France). Cycle thresholds (Ct) value will be manually set so that it intersects the exponential curve at its inflexion point. Any exponential signal observed between 0 and 40 Ct value will be considered as positive.”

**Comment:** It is unclear whether the additional indicators described in the study outcomes would come from the study results or from other sources. Some, like clinical signs of CAP, can be analysed from the study data, whereas others, while for others, like agent-specific hospitalization and fatality rates, the corresponding data is not collected in the CRF.

**Action/Answer:** Agree. The following sentence has been added in *Other data collected*: “A subgroup of patients or parents/legal guardians, randomly selected, was called back at least one month after inclusion to re-evaluate the outcome of included cases (hospitalization, death).”

**Comment:** The primary objective states that the PAFs will be estimated “using a combination of conventional methods and transcriptomics”, but it is unclear in the analysis plan if or how the transcriptomics data will be used to determine the PAFs and how these transcriptomics data will be analysed.

**Action/Answer:** Agree. This is indeed misleading. We removed this part of the sentence in the primary objective and added the following secondary objective: “compare microbiological and transcriptomic methods in estimating viral vs bacterial attributable fractions of LRTI”.
